# Topical niclosamide (ATx201) reduces *Staphylococcus aureus* colonization and increases Shannon diversity of the skin microbiome in atopic dermatitis patients in a randomized, double‐blind, placebo‐controlled Phase 2 trial

**DOI:** 10.1002/ctm2.790

**Published:** 2022-05-06

**Authors:** Anne Weiss, Emilie Delavenne, Carina Matias, Heimo Lagler, Daniel Simon, Ping Li, Jon U. Hansen, Teresa Pires dos Santos, Bimal Jana, Petra Priemel, Christine Bangert, Martin Bauer, Sabine Eberl, Alina Nussbaumer‐Pröll, Zoe Anne Österreicher, Peter Matzneller, Tamara Quint, Maria Weber, Hanne Mørck Nielsen, Thomas Rades, Helle Krogh Johansen, Henrik Westh, Wooseong Kim, Eleftherios Mylonakis, Christian Friis, Luca Guardabassi, John Pace, Carina Vingsbo Lundberg, Fatima M'Zali, Pascal Butty, Nikolaj Sørensen, Henrik Bjørn Nielsen, Rasmus Toft‐Kehler, Emma Guttman‐Yassky, Georg Stingl, Markus Zeitlinger, Morten Sommer

**Affiliations:** ^1^ UNION Therapeutics Hellerup Denmark; ^2^ Department of Medicine 1, Division of Infectious Diseases and Tropical Medicine Medical University of Vienna Wien Austria; ^3^ Department of Bacteria, Parasites and Fungi Statens Serum Institut Copenhagen Denmark; ^4^ Department of Veterinary and Animal Sciences University of Copenhagen Frederiksberg C Denmark; ^5^ Department of Pharmacy University of Copenhagen Copenhagen Denmark; ^6^ Department of Dermatology Medical University of Vienna Wien Austria; ^7^ Department of Clinical Pharmacology Medical University of Vienna Wien Austria; ^8^ Department of Clinical Microbiology Rigshospitalet Copenhagen Denmark; ^9^ Department of Clinical Medicine, Faculty of Health and Medical Sciences University of Copenhagen Copenhagen Denmark; ^10^ Hvidovre Hospital Hvidovre Denmark; ^11^ Warren Alpert Medical School of Brown University, Division of Infectious Diseases, Rhode Island Hospital Providence Rhode Island USA; ^12^ University of Bordeaux Bordeaux Cede; ^13^ CEVA Santé Animale Libourne France; ^14^ Clinical Microbiomics Copenhagen Denmark; ^15^ Icahn School of Medicine at Mount Sinai New York New York USA; ^16^ Novo Nordisk Foundation for Biosustainability Technical University of Denmark Lyngby Denmark

**Keywords:** bench‐to‐bedside, dermatology, microbiome, small molecule

## Abstract

**Background:**

In patients with atopic dermatitis (AD), *Staphylococcus aureus* frequently colonizes lesions and is hypothesized to be linked to disease severity and progression. Treatments that reduce *S. aureus* colonization without significantly affecting the skin commensal microbiota are needed.

**Methods and findings:**

In this study, we tested ATx201 (niclosamide), a small molecule, on its efficacy to reduce *S. aureus* and propensity to evolve resistance in vitro. Various cutaneous formulations were then tested in a superficial skin infection model. Finally, a Phase 2 randomized, double‐blind and placebo‐controlled trial was performed to investigate the impact of ATx201 OINTMENT 2% on *S. aureus* colonization and skin microbiome composition in patients with mild‐to‐severe AD (EudraCT:2016‐003501‐33). ATx201 has a narrow minimal inhibitory concentration distribution (.125–.5 μg/ml) consistent with its mode of action – targeting the proton motive force effectively stopping cell growth. In murine models, ATx201 can effectively treat superficial skin infections of methicillin‐resistant *S. aureus*. In a Phase 2 trial in patients with mild‐to‐severe AD (*N* = 36), twice‐daily treatment with ATx201 OINTMENT 2% effectively reduces *S. aureus* colonization in quantitative colony forming unit (CFU) analysis (primary endpoint: 94.4% active vs. 38.9% vehicle success rate, *p* = .0016) and increases the Shannon diversity of the skin microbiome at day 7 significantly compared to vehicle.

**Conclusion:**

These results suggest that ATx201 could become a new treatment modality as a decolonizing agent.

## Graphical abstract

ATx201 reduces S.aureus in vitro and in vivo with a narrow MIC distribution consistent with its mode of action targeting the proton motive force effectively stopping cell growth. In a Phase 2 trial in patients with mild‐to‐severe AD, twice‐daily treatment with ATx201 OINTMENT 2% effectively reduces S. aureus colonization and increases the Shannon diversity of the skin microbiome at day 7 significantly compared to vehicle. Thus, ATx201 could become a new treatment modality as a decolonizing agent.

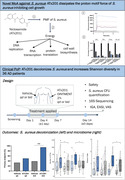

## 1 INTRODUCTION

Atopic dermatitis (AD) is a chronic inflammatory disease with a prevalence of 2%–10% in adults in developed countries.^1^ AD is a multifactorial disease with a complex interplay of immunological mechanisms, predominantly Th2/Th22 immune responses, and an altered epidermal skin barrier, characterized by trans‐epidermal water loss and epidermal hyperplasia.^2^, ^3^ Patients with AD are prone to bacterial colonization by *Staphylococcus aureus* on both lesional and non‐lesional skin.^4^, ^5^, ^6^ Indeed, more than 90% of patients with AD are colonized with *S. aureus* and the density of *S. aureus* on acutely inflamed AD lesions is generally more than 1000‐fold higher than on non‐lesional AD skin and peaks during disease flares.^5^, ^7^ The greater *S. aureus* colonization and proliferation probably results from a combination of a compromised functional epidermal barrier with reduced lipid content, increased adherence potential because of augmented fibronectin and fibrinogen in the stratum corneum, diminished immune recognition and impaired antimicrobial peptide production in patients with AD.^8^, ^9^, ^10^, ^11^, ^12^

In turn, *S. aureus* colonization is a significant factor in the pathogenesis of AD that exacerbates the disorder.^5^, ^11^ *S. aureus* secretes toxins that act as super‐antigens and exogenous protease inhibitors that further damage the epidermal barrier and potentiate allergen penetration that trigger multiple inflammatory reactions even in the absence of overt infection.^11^ *S. aureus* enterotoxins provoke the generation of enterotoxin‐specific IgE and thereby increase inflammation in AD.^13^ These enterotoxins interact directly with MHC II and the beta chain of the T‐cell receptor to induce an antigen‐independent proliferation of majorly type 2 T cells.^13^ Secretion of alpha toxin, a destructive pore‐forming toxin often found in the lesions of patients with severe AD, is primarily associated with keratinocyte death, which in turn correlates to increased exposure to Th2 cytokines.^14^, ^15^, ^16^ Expression of delta toxin by *S. aureus*, a phenol‐soluble modulin peptide, has been described to cause lysis‐independent mast cell degranulation and was also found in lesions of AD patients.^6^, ^9^

In recent studies, it was hypothesized that *S. aureus* relative abundance was further linked to the skin microbiome dysbiosis in patients with AD as shifts in microbial diversity and load of *Staphylococcus* were correlated to specific AD disease states and disease progression.^7^ The abundance of *Staphylococci*, specifically *S. aureus*, was shown to be greater and the Shannon diversity lower during disease flare compared to baseline or post‐treatment/resolving flares.^7^ Thus, *S. aureus* and the skin microbiome represent a promising target for the treatment of AD by addressing the ’outside‐in’ mechanism of the disease, although the causality of *S. aureus* decolonization and AD outcomes remains to be elucidated.^2^ Indeed, antibiotics such as fusidic acid or mupirocin are commonly prescribed to AD patients to address the microbial component of the disease. Unfortunately, these antibiotics exhibit major drawbacks. Worldwide resistance rates of *S. aureus* towards fusidic acid and mupirocin range in AD patients from 2.6% to 41% and 4% to 41%, respectively, and resistance towards fusidic acid evolves rapidly during treatment.^17^, ^18^, ^19^, ^20^, ^21^, ^22^, ^23^, ^24^, ^25^, ^26^, ^27^, ^28^, ^29^, ^30^

To exploit recent insights regarding the importance of *S. aureus* in AD, microbiome therapies are emerging to the clinic with the objective to reduce the abundance of *S. aureus* and increase microbial diversity by introducing one or more beneficial commensal strains. It was reported that transplantation of *Staphylococcus hominis* and *Staphylococcus epidermidis* strains reduced the bacterial load of *S. aureus* on the skin of AD patients, through exerting antimicrobial activity towards *S. aureus*.^31^ Another study demonstrated that *Bacillus subtilis* lipopeptides eliminate *S. aureus* by inhibiting its quorum‐sensing ability.^32^ The presence of Esp‐secreting *S. epidermidis* was also shown to eliminate the nasal colonization of *S. aureus* by diminishing the biofilm formation of *S. aureus*.^33^ Although therapies based on addition of living bacteria represent a promising therapeutic approach, challenges include the affordable manufacturing, formulation and long‐term stability of such therapeutics. Furthermore, it remains unclear whether the use of transplants of specific commensal bacteria can eliminate resistant strains on the human AD skin, such as methicillin‐resistant *S. aureus* (MRSA). Thus, a small molecule with a narrow‐spectrum effect, including *S. aureus*, could represent an attractive alternative for the treatment of AD.

We have repurposed niclosamide, a marketed oral chewable tablet to treat tapeworm infections, towards a topical decolonizing agent against *S. aureus*. Previous studies have demonstrated the potent antibacterial effect of niclosamide against *S. aureus* and MRSA in vitro.^34^, ^35^, ^36^, ^37^ A study of Gwisai et al. found that niclosamide inhibits a maturation of *S. aureus* biofilm, which was further validated by Torres et al., and niclosamide‐coated surfaces exhibited potent anti‐*S. aureus* activity.^34^, ^35^ Rajamuthiah et al. demonstrated that niclosamide exerts bacteriostatic killing kinetics and is effective against several drug‐resistant clinical strains, including MRSA, vancomycin‐resistant, linezolid‐resistant and daptomycin‐resistant strains.^36^

In this study, we examined the potency and the spectrum of ATx201 (niclosamide) in preclinical models and further analysed its propensity for resistance evolution. The impact of ATx201 OINTMENT 2% on the skin microbiome and load of *S. aureus* was then assessed in a clinical trial in patients with mild‐to‐severe AD.

## 2 METHODS AND MATERIALS

### 2.1 In vitro studies

#### 2.1.1 Bacterial strains and reagents

The clinical strains MRSA 43484 and 88779 were isolated from patients with skin abscesses and were obtained from UNION therapeutics A/S. The laboratory strains of *S. aureus* used in this study (ATCC 29213, ATCC 25293, NCTC 8325, and JE2) were obtained from the Department of Veterinary Disease Biology at the University of Copenhagen. *S. aureus* RN4220 and its mutator derivative RN4220 ΔmutS were kindly provided by A. J. O'Neill (University of Leeds, UK).

All strains were cryopreserved in Brain Heart Infusion broth supplemented with glycerol 15% (v/v) at −80°C and cultivated aerobically at 37°C in nutrient agar base no. 2 supplemented with calf blood 5% (v/v) (Oxoid, Basingstoke, UK). All bacterial cultures were grown in Mueller–Hinton broth (MHB) (Oxoid, Basingstoke, UK). When appropriate, culture medium was supplemented with ATx201 (Niclosamide; CAS: 50‐65‐7), rifampicin, fusidic acid, mupirocin, and retapamulin (Sigma‐Aldrich) and the pH adjusted to different values with NaOH and HCl solutions of different concentrations (.1, 1, 2 and 5 M).

#### 2.1.2 Susceptibility analysis of ATx201 and comparators

Minimal inhibitory concentrations (MICs) were determined by the broth microdilution method according to the recommendations of The Clinical & Laboratory Standards Institute.^38^

#### 2.1.3 Time‐kill kinetics of ATx201

For the time‐kill kinetics of ATx201, overnight cultures of strains MRSA 43484 and RN4220 were diluted in MHB to an OD_600_ = .1 and ∼1.25 × 10^6^ cells from these starter cultures were inoculated into flasks containing 25 ml of MHB (∼5 × 10^4^ CFU/ml) with different concentrations of ATx201 (drug‐free, 1 × MIC, 1.5 × MIC and 4 × MIC). The flasks were then incubated at 37°C, and colony forming unit (CFU) counts were performed at time points 0, 24, 48, and 72 h by spotting three 10‐μl drops of the diluted cultures on blood agar (BA) plates. All BA plates were incubated at 37°C and colonies were counted 24 h later. Data were obtained in two independent experiments.

#### 2.1.4 Bacterial growth inhibition study

MHB overnight culture of ATCC 29213 was sub‐cultured in fresh broth and grown up to OD_600_ = .3. Afterwards, freshly grown cells were distributed to separate tubes and treated with 1 ×, 4 × or 10 × MIC of ciprofloxacin, rifampicin, erythromycin, vancomycin and ATx201. Untreated control was also included. Cells were further grown at 37°C and optical density was recorded at 1‐h intervals at 600 nm and plotted against time.

#### 2.1.5 Molecule biosynthesis rate determination assay

Macromolecule biosynthesis experiments were performed as described by Ling et al.^39^ Briefly, *S. aureus* ATCC 29213 overnight culture was sub‐cultured at 1:100 in minimal media (.02‐M HEPES, .002‐M MgSO_4_, .0001‐M CaCl_2_, .4% succinic acid, .043‐M NaCl_2_, .5% (NH_4_)_2_SO_4_) supplemented with 5% tryptic soy broth and grown to OD_600_ = .2. Cells were pelleted down by centrifugation and resuspended in fresh media followed by incubation for 15 min (for cell wall synthesis 30 min) with negative control dimethyl sulfoxide (DMSO), ATx201, carbonyl cyanide m‐chlorophenylhydrazone (CCCP) or positive control of DNA, RNA, protein or cell‐wall synthesis inhibitors ciprofloxacin, rifampicin, erythromycin and vancomycin, respectively. The MICs of *S. aureus* ATCC 29213 for ciprofloxacin, rifampicin, erythromycin and vancomycin were found to be .25, .003, .5 and 1 μg/ml, respectively. After incubation, cells were pulse labelled individually by radiolabeled DNA, RNA, protein and cell‐wall synthesis precursors (50 μCi) ^3^H‐thymidine, (5 μCi) ^3^H‐uridine, (50 μCi) ^3^H‐leucine and (5 μCi) ^3^H‐glucosamine hydrochloride per ml, respectively, for 20 min (for cell‐wall synthesis cells 10 min). Finally, samples were precipitated with an equal volume of cold 30% TCA. Precipitates were membrane filtered and subsequently washed twice with cold 15% TCA and twice with water. Filters were air dried and stored in 10‐ml scintillation vials. Finally, 3‐ml scintillation fluid was added to each vial and a ^3^H count was taken in a Beckman Coulter LS6500 liquid scintillation counter for 1 min. Radioactive count of control (DMSO) treated sample was considered 100% precursor incorporation in macromolecule synthesis. Accordingly, the percentage rate of other treated samples was calculated. Data were obtained in two independent experiments.

#### 2.1.6 DiSC3(5) fluorescence‐based membrane potential study

MHB overnight cultures of ATCC 29213 and MRSA 43484 were sub‐cultured 1:100 in fresh MHB media and grown to OD_600_ = .2. Grown cells were diluted to OD_600_ = .1 in MHB and subsequently labelled with 1 μM 3,3′‐dipropylthiadicarbocyanine iodide (DiSC3(5)). The fluorescence spectra of labelled cells were taken in a microplate reader (Synergy H1, Holm & Halby) at Ex/Em wavelengths 622/670 nm over time. After reading of initial stable DiSC3(5) spectra, labelled cells were treated with DMSO, ATx201, CCCP, Vancomycin, Ciprofloxacin, Rifampicin or Erythromycin and change of DiSC3(5) fluorescence was recorded over time.

#### 2.1.7 Cytoplasmic pH change measurement

MHB overnight culture of ATCC 29213 was sub‐cultured 1:100 in fresh media and grown to OD_600_ = .2 and subsequently incubated with 10 μg/ml of 2′,7′‐bis(2‐carboxyethyl)‐5(6)‐carboxyfluorescein acetoxymethyl ester (BCECF‐AM), a pH sensitive cytoplasmic specific dye, for 30 min at 30°C. Incubated cells were pelleted down by centrifugation and resuspended in phosphate buffer saline (1.37‐M NaCl, 27‐mM KCl, 100‐mM Na_2_HPO_4_, 18‐mM KH_2_PO_4_, pH 7.4) supplemented with 25‐mM glucose. Subsequently the cells were adjusted to OD_600_ = .2. The cell suspension was kept at room temperature for 1 h to equilibrate the cytoplasmic pH. BCECF fluorescence of labelled cells was recorded in a PerkinElmer LS50B fluorescence spectrometer at Ex/Em wavelengths 485/520 nm using the time drive application of FLWINLAB software. After initial stable reading of BCECF spectra, labelled cells were treated with DMSO, ATx201, nigericin and CCCP individually. The changes of BCECF fluorescence upon treatments (FU 2.8 min – FU .2 min) were plotted. Data were obtained in two independent experiments.

#### 2.1.8 Estimation of spontaneous mutation rates

A fluctuation analysis was adapted from Rosche et al. and Gerrish et al.^40^, ^41^ Approximately 2.5 × 10^4^ cells from late‐exponential phase cultures were inoculated into 250 ml of MHB (10^2^ CFU/ml) and distributed into 24 tubes containing 10 ml of MHB with ∼10^3^ cells each. These were incubated at 37°C with agitation (125 rpm) and grown to late stationary phase. After incubation, ∼10^9^ cells were spread onto Mueller Hinton agar plates containing .25, .375 and 1 μg/ml of ATx201 by centrifuging 1 ml of culture for 3 min at 5000 rpm, partially discarding the supernatant and resuspending the pellet in the remaining media. The rate of mutational resistance to rifampicin was determined as a control, whereas the rates of mutational resistance to fusidic acid, mupirocin and retapamulin were determined for comparison purposes (∼10^8^ cells). The number of resistant colonies was counted after 48 h of incubation at 37°C. The average final number of cells (Nf) was obtained from four random cultures by spotting triplicate 10 μl‐drops of 1:10 serial dilutions in BA. The most likely number of mutations per culture (*m*) that gave rise to the distribution of mutants observed in the parallel cultures was estimated with a maximum likelihood method using the recursive formula implemented by Gerrish et al.^41^ The mutation rate (*μ*) (number of mutations per cell per division) was then calculated by dividing *m* by Nf. The mutation rate estimates represent the mean value of two independent experiments. The frequency of mutants (*F*) was obtained by multiplying the average number of colonies from the 24 cultures by the volume factor and dividing the resulting value by the Nf. Strains RN4220 and RN4220 ΔmutS were used to validate the fluctuation assay.

#### 2.1.9 Susceptibility testing after 40‐day passage

The in vitro development of resistance to ATx201 were carried out by subjecting MRSA 16115, isolated from dog otitis in Poland and 38127, isolated from hospital in France (QC strain: ATCC 29213) in liquid broths to serial passages to sub‐inhibitory concentrations of ATx201 (1/4 MIC) for 40 passages. After every 10 passages, the MIC of ATx201 was determined. The MICs were realized by broth dilution according to EUCAST guidelines.

### 2.2 Mouse studies

All studies comprising animals were conducted in accordance with the National Committee of Animal Ethics.

#### 2.2.1 Bacterial strains and mice

Strains MRSA 43484, MRSA 88779 and MRSA 86446, obtained from Statens Serum Institute, Copenhagen, Denmark and representing different resistance and genomic backgrounds, were selected to inoculate the mice. Bacterial suspensions for inoculation of mice were prepared from overnight cultures on 5% BA plates immediately before inoculation by suspending colonies in sterile .9% saline to 9 log_10_ CFU/ml. Female BALB/c mice, 10–12 weeks old (Taconic, Ejby, Denmark) were housed at Statens Serum Institut. All animal experiments were approved by the National Committee of Animal Ethics, Denmark. License No. 2013‐15‐2935‐00900.

#### 2.2.2 Experimental skin wound infection model

The experiments were conducted essentially as described by Vingsbo et al.^42^ Female BALB/c mice, 10–12 weeks old (Taconic, Ejby, Denmark), were anaesthetized and shaved on a 2–3‐cm^2^ skin area of the lower back. A disposable 7‐mm dermal curette (Integra, York, PA, USA) was used to induce a minor superficial skin wound on a 1‐cm^2^ area, which was inoculated with ∼7 log_10_ CFU of bacteria. Four days after inoculation and treatment, the mice were sacrificed and the entire infected skin area with underlying tissue was cut out and homogenized in 1 ml of saline using gentleMACS™ C‐tubes (Miltenyi Biotec, Lund, Sweden) on a Dispomix® Drive (Medic Tools AG, Zug, Switzerland) running at 4000 rpm for 10 s. Tissue homogenates were serially diluted 10‐fold in saline containing .1% Triton X‐100 (T‐8787; Sigma‐Aldrich Inc., St Louis, MO, USA). Then, 20‐μl spots were applied in duplicate to NaCl agar supplemented with polymyxin B (SSI Diagnostica). In addition, 100 μl of the 10^−2^ dilution was spread on a separate plate to increase sensitivity and minimize the risk of a false negative due to carry over of high drug concentrations from the wound. Plates were incubated at 35°C in ambient air for 20–48 h.

#### 2.2.3 Formulation preparation of ATx201 for animal studies

The ATx201 formulations used for the skin wound infection model were prepared at UNION therapeutics A/S. The comparators used were a Fucidin® and Bactroban®. Fucidin® is a commercial product registered in Denmark: (Fusidic acid 2%; LEO Pharma A/S, Ballerup, Denmark). Bactroban® is the commercial product registered in Denmark (Bactroban® nasal 2%, GSK).

#### 2.2.4 Treatment of skin infection

Mice were treated twice‐daily at approximately 24, 30, 48, 54, 72 and 78 h after inoculation by applying topically .05 ml of the ATx201 formulation or the comparators to the lesion area. Skin samples were collected the day after completed treatment for CFU determination. Carry‐over effects were minimized by performing the CFU determinations >18 h after the last treatment and homogenizing, diluting and plating the samples immediately.

### 2.3 Phase 2 study

#### 2.3.1 Trial design

This Phase 2, prospective, randomized, double blind, placebo‐controlled single‐centre trial was designed to evaluate the safety and efficacy of ATx201 OINTMENT 2% (drug substance: niclosamide) as a cutaneous decolonizing agent in AD patients versus vehicle (DECOLAD Part 2 EudraCT 2016‐003501‐33, NCT03009734). The study was performed from December 2016 until March 2018 in the Department of Clinical Pharmacology at the Medical University of Vienna and was conducted according to the provisions of the Declaration of Helsinki and its current amendments, and the International Conference on Harmonization Guidelines for Good Clinical Practice. The study was approved by the ethics commission of the Medical University of Vienna. The trial results are reported according to the CONSORT guideline (Table S1).

#### 2.3.2 Participants

**FIGURE 1:**
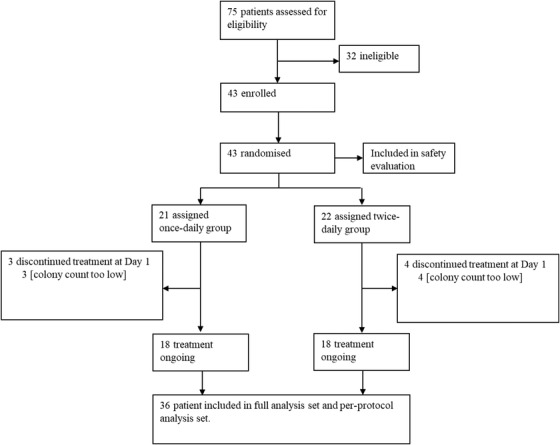
Trial profile

The study was composed of male and female subjects between 18 and 70 years of age with signed and dated informed consent obtained and diagnosed with AD (according to the Hanifin and Rajka criteria^43^) presenting as localized disease with three lesions. Further, patients with two individual lesions each covering an area between 10 and 200 cm^2^, having a lesional investigator's global assessment (lesional IGA) score between 1 and 4 and colonized by *S. aureus* with at least 1000 CFU/cm^2^, and a third localized lesion of area between 10 and 200 cm^2^ with a lesional IGA score between 1 and 4 were included. The full list of inclusion and exclusion criteria is provided in Table S2. The flow of participants is displayed in Figure 1.

#### 2.3.3 Interventions

The study consisted of a screening visit, a 7‐day treatment period, and an end‐of‐study visit on day 14. Patients with AD and colonized by *S. aureus* were treated once or twice‐daily on two defined treatment areas between 10 and 200 cm^2^ using either ATx201 OINTMENT 2% (active: niclosamide) or a matching vehicle (split‐body design). ATx201 OINTMENT 2% had to be dosed with a thin layer covering the entire lesion as instructed by the clinical personnel at the randomization visit. Only the first treatment application was performed by the investigator, the remaining applications were done by the patients. The visit and assessment schedule can be found in Table S3.

#### 2.3.4 Outcome

The primary objective of the study was to demonstrate the safety and tolerability of ATx201 OINTMENT 2% in patients with AD and to assess efficacy of ATx201 OINTMENT 2% in eradicating *S. aureus* compared to vehicle after 7 days of treatment. Treatment success as primary efficacy criteria was defined as a 100‐fold reduction in the *S. aureus* CFU/cm^2^ of skin lesions after 7 days of treatment. The secondary efficacy criterium was defined as the relative decrease of *S. aureus* CFU/cm^2^ on treated skin after 7 days treatment of the ATx201 OINTMENT 2% group, compared to vehicle and untreated area. The secondary objective of the study was to assess the impact of ATx201 OINTMENT 2% on the treated AD lesion and the systemic exposure of ATx201. To assess the AD lesions, the lesional IGA score, a modified eczema area and severity index (EASI) score, and lesional visual analogue scale (VAS) pruritus score, focused only on the target lesion, were utilized for area assessment.

The modified EASI assesses the target lesion by evaluating the four signs of eczema^44^: redness (erythema, inflammation); thickness (induration, papulation, swelling – acute eczema); scratching (excoriation); lichenification (lined skin, prurigo nodules – chronic eczema). The intensity of each sign is graded using a 4‐point scale (0 = absence; 1 = mild; 2 = moderate, 3 = severe). The modified EASI score is the sum of the intensity scores for each sign (0–12 points).

The lesional IGA score was defined as 0: clear (no inflammatory signs); 1: almost clear (just perceptible erythema, just perceptible papulation/infiltration); 2: mild disease (mild erythema and mild papulation/infiltration); 3: moderate disease (moderate erythema, moderate papulation/infiltration); 4: severe disease (severe erythema, severe population/infiltration).

All safety data were listed for subjects (patients) in the safety population. An adverse event (AE) was defined as any untoward adverse change from the subject's baseline condition, that is, any unfavourable and unintended sign, including an abnormal laboratory finding, symptom or disease which is considered to be clinically relevant by the physician that occurs during the course of the study, whether or not considered related to the study drug. The definitions used to grade the relationship of an AE to the study drug are displayed in Table S4.

#### 2.3.5 Randomization and masking

For each patient, two treatment areas were selected for treatment (ATx201 OINTMENT 2% or vehicle), and patients were randomized to receive active left and vehicle right or vice versa (split‐body design). A third (untreated) lesion was selected. An additional grouping factor was once‐daily versus twice‐daily application. If possible, target lesions were to be chosen symmetrically on the left and on the right side of the body, respectively. However, if there were no target lesions on both sides of the body, the investigator could choose two target lesions on the same side of the body. The randomization list was generated and maintained by an external vendor according to the study design. Once generated, the randomization code was final and not modifiable. Sealed envelopes (Code Break Envelope) contained treatment assignments. Patients were randomized according to a list with the respective IP number for the patient. Each patient was assigned a number corresponding to the order of entering the study and thus was assigned to the treatment group according to a block randomization schedule. All personnel involved in the study, including the investigators, site personnel, and the sponsor's staff (e.g. CRA/Monitor), were blinded to the IP codes. The treatments were blinded to both patients and physicians. The placebo formulation of the placebo product was identical to the active formulations in terms of looks and feel.

#### 2.3.6 Sample collection

Skin swabs were obtained at screening visit (up to five lesions), Baseline (day 1) and end‐of‐treatment (EOT) (day 7) for quantitative culture of *S. aureus* using a sterile cotton swab dipped into a buffer of .1% Triton X‐100 in .075‐M phosphate buffer, pH 7.9. A template of 6.4 cm^2^ was streaked with a cotton swab for 10 s and solubilized in 1‐ml buffer. This sample collection procedure has been shown to maximize the recovery of *S. aureus* from skin of AD patients.^45^ One part of the buffer was used for a quantitative culture of *S. aureus* and the other part for quantification of microbiome diversity.

#### 2.3.7 Quantitative culture of *S. aureus*

All collected liquid samples were submitted and processed immediately. The swap buffer solution was vortexed mildly to release bacteria. Samples of the skin swab solution were serially diluted and streaked on ChromID agar plates. After incubation at 37°C over 24 h the *S. aureus* specific colonies were counted and the amount of bacterial load was described as CFU per cm^2^ of the investigated AD skin area. The cut‐off value for definition of colonization of lesions with *S. aureus* was defined as 1000 CFU/cm^2^.

#### 2.3.8 Quantification of skin microbiome diversity

Skin samples obtained at Baseline and EOT were further used for the quantification of skin microbiome diversity (α‐ and β‐diversity) by 16S rDNA sequencing using an established methodology.^7^ The α‐diversity of the samples was assessed by counting the number of OTUs (operational taxonomical units, similar to species richness) and calculating the Shannon index.

To measure the β‐diversity, we used generalized UniFrac^46^ which is an intermediate between weighted and unweighted UniFrac (*α* = .5). A detailed description can be found in the supplementary material.

#### 2.3.9 Pharmacokinetic analysis

Blood samples were drawn 1, 2, 4, 6 and 8 h after treatment. Niclosamide concentration in human EDTA plasma (K_2_EDTA) samples were analysed by LC–MS/MS. Individual and descriptive statistics of systemic exposure of ATx201 OINTMENT 2% concentration‐time data were conducted by noncompartmental model of Phoenix WinNonlin version 6.3 or higher (Pharsight Corporation, St. Louis, MO). An analysis of covariance was performed by using SAS PROC GLM.

### 2.4 Statistical analysis

#### 2.4.1 In vivo model

CFU data was log_10_ transformed prior to analysis. Samples that were CFU negative (i.e. below the lower detection limit of 50 CFU) were assigned a CFU value of 50. ANOVA and Dunnett's multiple comparison tests were used to compare log_10_ CFU values for multiple groups. *p*‐Values <.05 were considered significant. All statistical comparisons were made using the software GraphPad Prism 6.00 for Windows, GraphPad Software, CA, USA.

#### 2.4.2 Phase 2 study

A formal sample size calculation was not performed and the sample size of 40 patients was regarded as sufficient to get preliminary information about safety and tolerability of the IP in different regimens. A treatment success of 70%–90% for ATx201 OINTMENT 2% and 10%–15% for vehicle was estimated. With ‘worst case’ assumptions, 18 patients per group were needed for a statistically significant difference of *p* <.025 in favour to ATx201 OINTMENT 2% with a power of 80%. Assuming a drop‐out rate of 10%, 20 patients per group (in total 40 patients) were needed to keep a global alpha of .05.

Statistical tests regarding the primary endpoint of the study were performed using a chi‐square test for dependent samples in SAS® Version 9.2 (Test of Symmetry according to Bowker). To keep a global alpha of .05 (two‐sided), each of the two hypotheses was tested at a significance level of *α* = .025, two‐sided. Secondary efficacy variables were presented using appropriate descriptive methods.

Statistical test for change in *S. aureus* abundance level (CFU) was performed using Wilcoxon test. Statistical tests regarding the microbiome analysis were performed using Wilcoxon test. Notwithstanding, statistical tests are defined in Section 3. Statistical tests were made using *R* (version 3.3 or greater).

#### 2.4.3 Role of funding sources

The funder of the study had no role in study design, data collection, data analysis, data interpretation or writing of the report. The corresponding author had full access to all the data in the study and had final responsibility for the decision to submit for publication.

## 3 RESULTS

### 3.1 ATx201 is a potent agent against *S. aureus* overcoming existing resistance mechanisms with dose‐dependent kill kinetics

**FIGURE 2:**
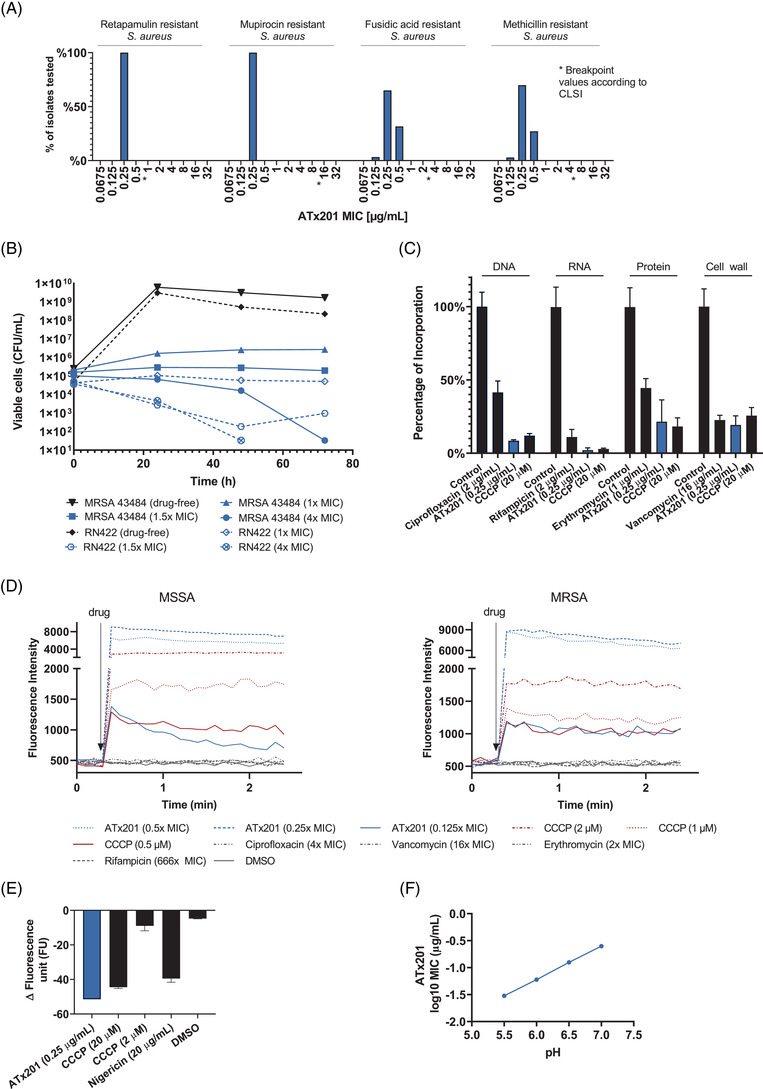
Time‐kill kinetics of ATx201 and its activity to dissipate the PMF of *Staphylococcus aureus*. (A) MIC distribution of ATx201 against all tested *S. aureus* strains depending of their resistance profile. (B) ATx201 killing kinetics for MRSA 43484 and *S. aureus* strain RN4220 in drug‐free MHB or MHB with different concentrations of ATx201. (C) Effect of ciprofloxacin, rifampicin, erythromycin, vancomycin, CCCP and ATx201 on macromolecule synthesis is presented by the percentage of incorporated DNA, RNA, protein and cell‐wall precursors relative to DMSO‐treated controls. Average percentage value of each treatment is presented with standard deviation. Antimicrobials with treatment concentration are labelled below the column of corresponding percentage value. DNA, RNA, protein and cell‐wall synthesis panels are designated above the columns by horizontal lines. Different fold‐MIC concentrations were used for different antibiotics given their MIC against MRSA strains. (D) Effect of ATx201, CCCP, dimethyl sulfoxide (DMSO) or ciprofloxacin, rifampicin, erythromycin, vancomycin treatments on DiSC3(5) fluorescence in ATCC29213 (left panel) and MRSA 43484 (right panel) are plotted as a function of time. 1 × MIC of ciprofloxacin, rifampicin, erythromycin, vancomycin and ATx201 are .25, .003, .5, 2 and .25 μg/ml, respectively. (E) BCECF fluorescence change upon ATx201, nigericin, CCCP or DMSO treatments. (F) Log_10_ MIC (μg/ml) of ATx201 and its dependence on pH. BCECF, 2′,7′‐bis(2‐carboxyethyl)‐5(6)‐carboxyfluorescein; CCCP, carbonyl cyanide m‐chlorophenylhydrazone; DiSC3(5), 3,3′‐dipropylthiadicarbocyanine iodide; MHB, Mueller–Hinton broth; MIC, minimal inhibitory concentration; MRSA, methicillin‐resistant *S. aureus*; PMF, proton‐motif force

ATx201 was identified in a previous high‐throughput screen as a compound with activity against MRSA USA300.^36^, ^47^ To assess the breadth of effect of ATx201 against a broad panel of clinical isolates, 206 clinically relevant *S. aureus* strains, including both MRSA (*N* = 118) and methicillin‐sensitive (*N* = 88) strains, were screened. The panel included strains resistant to commercialized comparators commonly used for the topical treatment of skin infections, such as fusidic acid (*N* = 60), mupirocin (*N* = 3), retapamulin (*N* = 1) and clindamycin (*N* = 13). The MIC of ATx201 ranged from .125 to .5 μg/ml against all *S. aureus* strains regardless of their resistance towards the comparators (Figure 2A and Table S5). The remarkable consistency in the MIC against diverse isolates of *S. aureus* suggests that growth might be inhibited through a novel mechanism of action.

To test whether ATx201 has a bacteriostatic or bactericidal effect, time‐kill curves against both methicillin‐resistant (MRSA 43484 = MRSA 01) and susceptible (RN422) strains of *S. aureus* were determined. ATx201 MICs were estimated after 18 h of incubation and were identical for both strains (Table S5). ATx201 exhibited bacteriostatic killing kinetics at 1 × and 1.5 × MIC, corresponding to .25 and .375 μg/ml, respectively. However, at 4 × MIC (1 μg/ml) ATx201 cleared the bacterial populations of RN422 and MRSA 43484 after 48 and 72 h, respectively (Figure 2B).

### 3.2 ATx201 arrests cell growth and inhibits specific biosynthetic pathways

To evaluate the kinetics of ATx201, the bacterial growth inhibition of exponentially growing cultures of *S. aureus* ATCC 29213 was treated with 1 ×, 4 × or 10 × MIC of ATx201 or known standard DNA, RNA, protein and cell‐wall synthesis inhibitors ciprofloxacin, rifampicin, erythromycin and vancomycin, respectively. Optical density at 600 nm was quantified over time for these different drug exposures. Immediate growth arrest was observed upon an addition of 1 × MIC ATx201 (Figure S1). In contrast, none of the comparators arrested cell growth immediately at 1 × MIC. Cell growth inhibition by 1 × to 10 × MIC ATx201 did not cause cell lysis (data not shown).

To further investigate the mechanism of action, the effect of ATx201 on macromolecular biosynthetic activity was characterized. The rate of DNA, RNA, protein and cell‐wall biosynthesis was studied using corresponding radiolabeled precursors along with a well‐characterized antibiotic inhibiting each corresponding biosynthetic activity. We found that ATx201 strongly inhibits all biosynthetic activities tested even with higher efficiency and at lower concentrations compared to the relevant specific antibiotic inhibitors (Figure 2C). These data suggest that ATx201 rapidly inhibits cellular metabolism upstream of the specific biosynthetic processes. In addition to the specific antibiotic inhibitors, we also tested CCCP a compound known to depolarize the cell membrane of bacteria. Notably, CCCP displayed a similar inhibition of the biosynthetic activity of the cell to ATx201, suggesting a similar mode of action (Figure 2C).

### 3.3 ATx201 dissipates the proton motive force and acts as a proton carrier

To study the impact of ATx201 on the proton motive force in *S. aureus*, we deployed a membrane‐specific fluorescence dye DiSC3(5), which localizes in the bacterial membrane in a proton motive force‐dependent manner such that its fluorescence increases upon a dissipation of the proton motive force.^48^ We found that DiSC3(5) fluorescence increased upon the treatment of DiSC3(5) labelled *S. aureus* ATCC 29213 or MRSA 43484 with ATx201 (Figure 2D). A similar observation was made when DiSC3(5) labelled cells were treated with CCCP. No change of fluorescence was observed upon treatment with equal volume of DMSO solvent or comparator antibiotics, such as vancomycin, erythromycin, rifampicin or ciprofloxacin. These observations suggest that ATx201 dissipates the proton motive force in *S. aureus*, including in MRSA strains, which is in alignment with previous studies.^49^ However, more studies are needed to elucidate the dose‐dependency of ATx201's effect on dissipating the proton motif force as we did not observe a clear dose–response in our studies.

Furthermore, to investigate the potential protonophore activity of ATx201 we used the dye BCECF‐AM, which is hydrolyzed by cytoplasmic esterases to the BCECF fluorophore^50^ and whose fluorescence depends on the cytoplasmic pH. Under normal conditions, the cytoplasmic pH lies within the range of 7.4–7.8 resulting in high BCECF fluorescence. However, the fluorescence of BCECF is reduced upon protonophore treatment due to influx of protons acidifying the cytosol.^51^ Indeed, treatment of BCECF labelled *S. aureus* ATCC 29213 cells with 20‐μg/ml nigericin, a protonophore H+/K+ anti‐porter, reduced BCECF fluorescence (Figure 2E and Figure S2). Also, a similar drop of fluorescence was observed when BCECF labelled *S. aureus* ATCC 29213 cells were treated with 20 μM of the protonophore CCCP. Treatment of BCECF labelled cells with .25‐μg/ml ATx201 also reduced BCECF fluorescence. Notably, the cytoplasmic acidification action of 20‐μg/ml nigericin and 20‐μM CCCP were close and comparable to .25‐μg/ml ATx201 (Figure 2E). No change of BCECF was observed when labelled cells were treated with 20‐μg/ml valinomycin, a K+ ionophore (data not shown).

If ATx201 (in solution pH of 7.4) acts as a protonophore on *S. aureus*, one would expect its potency to increase at acidic pH conditions. Indeed, it was found that the MIC of ATx201 decreased with decreasing pH (Figure 2F), which is in agreement with the hypothesis that ATx201 is a proton carrier that dissipates the PMF through acidification of the bacterial cytosol. Furthermore, it should be noted that the pH range of human skin is between 5 and 6, suggesting that ATx201 inhibitory activity is potentiated relative to the conditions normally used to determine MICs in vitro.

Overall, these observations suggest that ATx201 dissipates the proton motive force in *S. aureus*, including in MRSA strains, by reducing the cytoplasmic pH through a proton carrier activity. To the best of our knowledge, no approved antibacterial which is effective against *S. aureus* is known to function via this mode of action; accordingly, these data are consistent with ATx201 having a new mode of action.

### 3.4 *S. aureus* mutants resistant to ATx201 cannot be selected in the laboratory

A key consideration for the evaluation of the usefulness of any decolonizing agent is the emergence of single‐step mutations conferring resistance towards that agent. To assess the likelihood of emergence of spontaneous resistance mutations, a fluctuation assay was conducted for ATx201 and relevant comparators such as fusidic acid, mupirocin, retapamulin and rifampicin, using *S. aureus* MRSA 43484. At a concentration of 4 × MIC, we were unable to isolate any resistant mutants towards ATx201. In contrast, we found resistant mutants at 4 × MIC for all other tested comparators. The frequencies of resistant mutants of strain 43484 at 4 × MIC are 2.00 × 10^−8^, 7.90 × 10^−8^, 8.02 × 10^−7^ and 5.66 × 10^−9^ for mupirocin, retapamulin, fusidic acid and rifampicin, respectively (Table S6 and Table 1). These numbers are in agreement with previously published works^52^, ^53^ and correspond to 4 × MIC frequency values at least 1000 times higher than that of ATx201 at a concentration of 1 × MIC (*F* = 1.67 × 10^−11^). To further investigate the propensity of *S. aureus* to evolve resistance towards ATx201, we conducted a 40‐day serial passage experiment using two MRSA strains (MRSA 16115 and MRSA 38127). Following the 40‐day passaging, individual colonies were picked from the evolved populations and the MIC towards ATx201 was determined. Notably, the MIC towards ATx201 remained <.5 μg/ml for both MRSA 16115 and MRSA 38127 (Table 2), which additionally supports the low likelihood of resistance evolution of ATx201. Thus, these data highlight that the supply of mutations conferring resistance towards ATx201 is very low compared to currently used cutaneous antimicrobial agents.^54^

**TABLE 1:** Mutation rates (*μ*) and frequencies of resistant mutants (*F*) for ATx201 and other drugs in MRSA 43484

	*F*	*μ*	95% CI
ATx201 (1 × MIC = .25 μg/ml)	1.67 × 10^−11^	6.51 × 10^−12^	9.18 × 10^−13^–4.62 × 10^−11^
ATx201 (1.5 × MIC = .375 μg/ml)	<DL^a^	<DL	–
ATx201 (4 × MIC = 1 μg/ml)	<DL	<DL	–
Mupirocin (4 × MIC = 1 μg/ml)	2.00 × 10^−8^	2.46 × 10^−9^	1.98–3.06 × 10^−9^
Retapamulin (4 × MIC = .256 μg/ml)	7.90 × 10^−8^	7.50 × 10^−9^	6.46–8.72 × 10^−9^
Fusidic acid (4 × MIC = .256 μg/ml)	8.02 × 10^−7^	8.98 × 10^−8^	8.45–9.55 × 10^−8^

Abbreviations: MIC, minimal inhibitory concentration; MRSA, methicillin‐resistant *S. aureus*.

^a^ At ATx201 concentrations of 1.5 × and 4 × MIC, the general lack of growth indicates a very low frequency of ATx201‐resistant mutants (no colonies were observed on the plates), below the detection limit (DL) of our experimental set up, which could potentially detect 1 mutant in ∼10^9^ bacteria (corresponding to 1 ml of culture).

**TABLE 2:** MICs of ATx201 for *Staphylococcus aureus* strains exposed to 40 passages of sub‐inhibitory concentrations

	ATx201 (μg/ml)				
Strain	Parental strains	After 10 passages	After 20 passages	After 30 passages	After 40 passages
*S. aureus* ATCC 29213	.25	.125	.25	.25	.125
MRSA 16115	.06	.06	.06	.06	.06
MRSA 38127	.125	.125	.125	.25	.25

Abbreviations: MIC, minimal inhibitory concentration; MRSA, methicillin‐resistant *S. aureus*.

### 3.5 ATx201 is an effective decolonization agent in vivo

**FIGURE 3:**
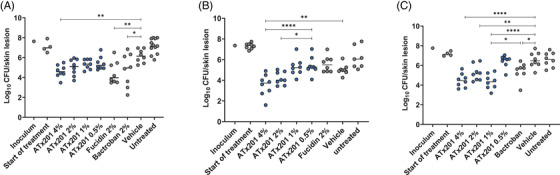
CFUs in the mouse superficial skin wound infection model. Mice were treated twice‐daily for 3 days with vehicle only, an ATx201 dose range or licensed products Fucidin or Bactroban following inoculation with (A) MRSA 43484 (FUS and MUP susceptible), (B) MRSA 88779 (FUS resistant) or (C) MRSA 86446 (MUP resistant). The data are representative of five (A) or two (B and C) independent experiments. **** = *p* < .0001, *** = *p* < .001, ** = *p* < .01, * = *p* < .05, Dunnett's multiple comparison test. CFU, colony‐forming unit; FUS, fusidic acid; MRSA, methicillin‐resistant *S. aureus*; MUP, mupirocin

To test ATx201 efficacy in vivo, a superficial skin infection model in BALB/c mice was developed.^42^ The model was prepared with three different MRSA strains, covering different resistance profiles, including resistance to fusidic acid and mupirocin. Experimental cutaneous formulations of ATx201, as well as commercial cutaneous formulations of fusidic acid and mupirocin, were applied twice‐daily for 3 days. We found that ATx201 significantly reduced the bacterial load of MRSA strains, including both fusidic acid and mupirocin resistant isolates. Treatment with ATx201 (4% w/w) against the type USA300 MRSA 43484 was equivalent to commercial fusidic acid (Fucidin®) and mupirocin formulations (Bactroban®) (Figure 3A). However, treatment with ATx201 (4% w/w) against the fusidic acid resistant isolate MRSA 88779 was superior to fusidic acid (*p* < .0001, ANOVA). Indeed, treatment with Fucidin® did not result in a significant reduction in MRSA 88779 load compared to the untreated mice (Figure 3B). ATx201 was also able to significantly reduce the bacterial load of the mupirocin resistant MRSA strain 86446 (Figure 3C). Surprisingly, despite an MIC of >256 μg/ml, the mean bacterial load of MRSA 86466 was also significantly reduced by 13.8‐fold following treatment with 2% w/w Bactroban® (*p* < .05, ANOVA). However, ATx201 (4% w/w) showed a statistically significant reduction in bacterial load compared to treatment with Bactroban® (*p* < .0001). In conclusion, these data demonstrate that cutaneous use of ATx201 is as effective as both fusidic acid and mupirocin when treating strains that are susceptible to fusidic acid or mupirocin. However, ATx201 displays a superior efficacy compared to the commercial formulations of fusidic acid and mupirocin when treating strains that are multidrug resistant.

### 3.6 ATx201 OINTMENT 2% is well tolerated with no safety issues reported in patients with AD

Following a Phase 1 study in healthy volunteers, in which three different cutaneous formulations of ATx201 were tested, the ATx201 OINTMENT 2% formulation was selected for further clinical testing. We studied the safety and efficacy of ATx201 OINTMENT 2% in patients with AD in a randomized, placebo‐controlled, double‐blinded, split‐body designed Phase 2 study (study design displayed in Figure S3). The trial was conducted in December 2016 until March 2018. Out of 43 patients that were randomized to ATx201 OINTMENT 2% once‐daily (qd) or twice‐daily (bid) and matching vehicle, 36 patients were included in the full analysis set (18 per treatment group). As prospectively defined in the inclusion criteria, seven patients were discontinued on day 1, due to *S. aureus* colony count below 1000 CFU/cm^2^. The participant flow is visualized in Figure 1.

Baseline characteristics and demographics were similar across the two dosage groups, once‐daily and twice‐daily (Table S7). Most patients had a Baseline lesional IGA score of 3 (58.3% in ATx201 OINTMENT 2% group, 55.6% in vehicle group). The mean Baseline modified EASI score was 7.81 in the ATx201 OINTMENT 2% group and 7.86 in the vehicle group, the mean Baseline lesional VAS Pruritus score was 4.52 and 4.5, respectively.

ATx201 OINTMENT 2% was well tolerated in both dosage groups as no safety issues were identified (overview of all AEs in Table S8). All systemic AEs reported in Table S8 were rated as ’not’ or ‘unlikely’ related to the study treatment and all recovered or were recovering without interrupting treatment (Table S4 contains detailed definitions of grading AE relationship). In contrast, only few local reactions were observed which all were graded as ‘possibly’, ‘probably’ or ‘related’. Local reactions were reported to be ‘mild’ or ‘moderate’ and also all fully recovered without need for interruption of treatment. This safety profile is in alignment with previous clinical trials conducted in healthy volunteers demonstrating ATx201 OINTMENT 2% as a well‐tolerated agent without any signs of irritation or sensitization at the site of administration and neglectable systemic exposure.^55^ ATx201 has been described as a mild mitochondrial uncoupler in human cells with a good safety profile, as mild uncoupling is tolerable in normal cells.^56^, ^57^ This dermatological safety and tolerability is further supported by a clinical study showing that topical application of ATx201 for 6 months did not result in side effects, as well as a murine study in which subcutaneous injection of ATx201 did not cause significant skin alterations in healthy mice.^58^, ^59^ The higher local reaction rate in our trial compared to the one reported in Podgore et al., investigating schistosomal infection could be explained by the fact that the skin of AD patients is abraded and thus more sensitive and prone to local reactions due to the lack of the stratum corneum and reduced barrier properties.^58^ However, we would like to point out that there was no difference in the number of administration site reactions between vehicle and active and we consider the number of these reactions low.

Furthermore, the mean systemic exposure of niclosamide following administration of ATx201 OINTMENT 2% was minimal with median peak exposure of 1.36 ng/ml (once‐daily) and 3.05 ng/ml (twice‐daily), which is substantially lower than the plasma levels reported with oral niclosamide at the approved dose (niclosamide chewable tablets; median *C*_max_ = 665–759 ng/ml^60^) of 2 g/day. As the chewable tablets of niclosamide have been used since the 1960s to treat tapeworm infections, even in pregnant women and children <2 years, and its use is considered safe (long‐term), treatment with ATx201 OINTMENT bears a very low risk of AEs resulting from systemic exposure.^61^

Treatment with ATx201 OINTMENT 2% for 7 days improved the clinical scores of the treated AD lesion (lesional IGA, modified EASI, lesional VAS), although not with significant difference between active and vehicle in neither of the two dosage groups (Figure S4 and Table S9).

### 3.7 ATx201 effectively reduces *S. aureus* abundance in AD lesions

**FIGURE 4:**
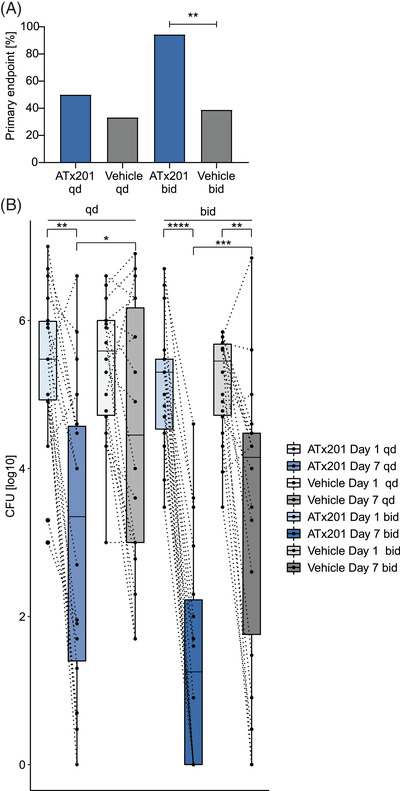
Impact of ATx201 OINTMENT 2% as a decolonizing agent in AD patients. (A) Share of patients achieving treatment success at day 7 receiving ATx201 OINTMENT 2% (ATx201) compared to vehicle, defined as a 100‐fold reduction in the *Staphylococcus aureus* CFU/cm^2^ per treatment arm. ** = *p* < .01, chi‐squared test. *N* = 18 per dosage group (split‐body design). (B) Change in abundance levels (CFUs) of *S. aureus* per dosage and treatment group. Paired samples are connected with a dotted line. *** = *p* < .001, * = *p* < .05, Wilcoxon test. bid = twice‐daily, qd = once‐daily. AD, atopic dermatitis

The primary efficacy endpoint in this study was eradication of *S. aureus* defined as a >100‐fold reduction in the *S. aureus* CFU/cm^2^ of sampled skin lesion on day 7 in the ATx201 OINTMENT 2% group compared to vehicle. For the once‐daily group, treatment with ATx201 OINTMENT 2% resulted in treatment success and 9 out of 18 patients (50.0%) at day 7, whereas vehicle showed a 33.3% (6 out of 18 patients) success rate, respectively. For the twice‐daily group, treatment with ATx201 OINTMENT 2% resulted in treatment success for 17 out of 18 patients (94.4%) at day 7, whereas vehicle demonstrated a 38.9% (7 out of 18 patients) success rate, respectively. The treatment success was significantly higher for active compared to vehicle at day 7 only in the twice‐daily group (chi‐squared test, *p* = .00157), but not in the once‐daily group (chi‐squared test, *p* = .18) (Figure 4). Untreated areas did not show a successful response in any dosage group at day 7 (2 out of 18 patients [11.1%], combined dosage groups). Furthermore, a significantly higher decrease of *S. aureus* CFU/cm^2^ was noted on skin areas treated with ATx201 OINTMENT 2% twice‐daily compared with a vehicle with ATx201 OINTMENT 2% bid‐treated areas having a ratio of Day1_CFU/cm2_ to Day7_CFU/cm2_ of 143 305.8 compared to vehicle‐treated areas having a mean ratio of 23 787.9 (*p* < .0024, Kruskal–Wallis test with Dunn's multiple comparison test). The decrease in *S. aureus* abundance in the vehicle‐treated groups could result from a moderate direct antibacterial activity of polyethylene glycol (PEG), a constituent of the vehicle, which has previously been described for *S. aureus*.^62^ Importantly, treatment with ATx201 OINTMENT 2% twice‐daily for 7 days significantly reduces *S. aureus* in skin lesions of AD patients compared to vehicle.

To investigate whether ATx201 would select for resistant mutants in this clinical study we isolated 26 isolates from 36 patients that had cultivatable *S. aureus* isolates at day 7. We determined the MIC for all isolates and did not observe any strains isolated on day 7 with increased MICs (>.5 μg/ml) towards ATx201 (Table S10). These data along with the in vitro assessments of resistance evolution support the hypothesis that ATx201 has a very low propensity to select for resistance.

### 3.8 ATx201 OINTMENT 2% increases the Shannon diversity of the skin microbiome of AD patients

To further assess the effect of ATx201 OINTMENT 2% on the skin microbiome, the α‐ and β‐diversity of ATx201 was investigated relative to a vehicle using 16S rDNA sequencing. The α‐diversity of the samples was assessed by counting the number of OTUs and calculating the Shannon index. The Shannon index does not only take the number of OTUs of a community into account but also includes their relative abundances. It will give a low diversity score to a community dominated by a few OTUs (even though the total number of OTUs may be high), whereas communities where many different OTUs have similar abundances will receive a high diversity score.

**FIGURE 5:**
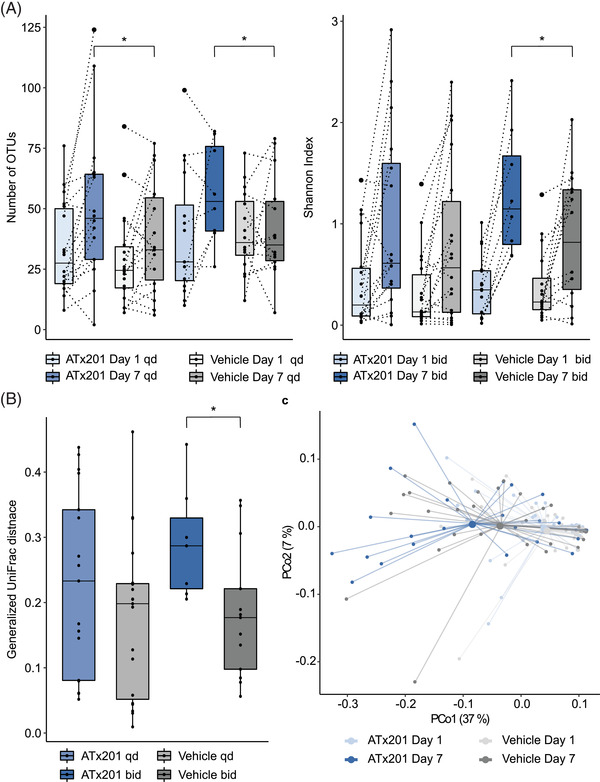
Effect of ATx201 OINTMENT 2% on the skin microbiome diversity compared to vehicle. (A) Boxplot of the number of OTUs and Shannon index per dosage group and visit. α‐Diversity was measured by counting the number of OTUs and calculating the Shannon Index. * = *p* < .05, Wilcoxon test. (B) Boxplot of the generalized UniFrac distance as measurement for β‐diversity between day 1 and day 7 samples within a subject. * = *p* < .05, Wilcoxon test. (C) PCoA plots of samples by treatment group (once‐ and twice‐daily combined) and time of sampling using generalized UniFrac distances calculated from OTU abundances. Paired samples are connected with a dotted line. bid = twice‐daily, qd = once‐daily. OTUs, operational taxonomic units; PCoA, principal coordinate analysis

The α‐diversities of the lesions treated with ATx201 OINTMENT 2% and vehicle were similar at baseline for the once‐daily (Wilcoxon test, *p*_OTUs_ = .3517, *p*_Shannon_ = .5619) and twice‐daily treatment group (Wilcoxon test, *p*_OTUs_ = .7422, *p*_Shannon_ = .5469). Treatment with ATx201 OINTMENT 2% for 7 days twice‐daily resulted in a significant increase of the α‐diversity relative to vehicle which was reflected in a significantly higher number of OTUs and a significantly increased Shannon index relative to vehicle (Figure 5A; Wilcoxon test, *p*_OTUs_ = .0142, *p*_Shannon_ = .0234). However, the α‐diversity also increased with vehicle after 7 days compared to baseline. This might result from a moderate direct antibacterial activity of PEG as described in the previous paragraph. However, it should be noted that treatment with ATx201 OINTMENT showed superior anti‐ staphylococcal effects compared to vehicle in all our studies.

The β‐diversity is a measure of dissimilarity in the taxonomic composition between samples which was analysed by generalized UniFrac.^46^ The change in microbiome community composition at day 7 compared to baseline was significantly greater for the ATx201 OINTMENT 2% twice‐daily treatment group compared to vehicle (Figure 5A; Wilcoxon test, *p* = .0234). Moreover, the compositional plot displays that ATx201 OINTMENT 2% twice‐daily significantly decreased the abundance of *S. aureus*, which is in alignment to the culture data obtained in this study considering the direction of the change. It further appears that other genera increase in abundance (Figures S5–S7). Overall, these data highlight that treatment of AD lesions with ATx201 reduces the abundance of *S. aureus* and increases the skin Shannon diversity significantly compared to vehicle.

## 4 DISCUSSION

In this study, we describe ATx201 as a potent compound against *S. aureus*, including drug‐resistant strains in vitro and in a murine skin infection model. We find that ATx201 inhibits *S. aureus* by dissipating the proton motive force through its protonophore activity leading to a rapid shutdown of bacterial biosynthesis and subsequently bacterial growth in a pH‐dependent manner. Targeting physical properties of the bacterial membrane has been described to reduce the likelihood of resistance evolution and benefit the elimination of persistent colonization.^63^ Consistent with this, we find that ATx201 has undetectable *de novo* resistance evolution strongly differentiating ATx201 from current cutaneous agents.

We showed that AD patients receiving ATx201 OINTMENT 2% twice‐daily had a significant reduction in the abundance of *S. aureus* and increasing Shannon diversity of skin microbiome compared to vehicle after 7 days. However, the impact of ATx201 on the healthy skin microbiota and specificity of ATx201's effect to *S. aureus* remains to be fully elucidated. Accordingly, higher resolution metagenomics and longer treatment duration would be needed along with approaches to quantitate whether increases in Shannon diversity result primarily from a reduction in *S. aureus* or a regrowth of the commensal microbiota.

Interestingly, throughout all microbiological assessments in the in vivo studies and clinical trial the vehicle component showed a tendency towards a mild antibacterial effect, although not significant in all assessments. We hypothesize that this might be due to the PEG excipient in the formulation as mentioned previously in‐line. Although it might be insightful to elucidate the degree of effect and the potential of synergistic action of active with the vehicle from a mechanistic angle, we have shown that ATx201 is statistically superior to vehicle in all studies with microbiological assessments underlining that the vehicle is not pivotal to the overall effect.

Although reduced *S. aureus* abundance and increased microbiome diversity have been linked to improved AD disease states, in our study, ATx201 qualitatively improved AD lesions during 7 days of twice‐daily treatment.^7^ We expect an extended treatment regimen would be required to further improve AD symptoms.

Although complete removal of *S. aureus* is preferable with a newly developed antibiotic, we consider that ATx201 provides a very differentiated profile to conventional antibiotics that typically are less selective. In contrast to fusidic acid, which has been demonstrated to select for resistant *S. aureus* during treatment AD patients,^27^ ATx201 does not seem to select readily for resistance in clinical use. Accordingly, ATx201 might be particularly well suited for longer term application in AD patients. Accordingly, as AD patients are a reservoir for MRSA/*S. aureus* ^4^, ^15^, ^64^, ^65^, ^66^ ATx201 could also be used to reduce *S. aureus* in such patients to diminish the risk of transmitting pathogens to the public as well as contracting serious infections.^67^, ^68^, ^69^ Yet, the patient groups targeted by ATx201 could be extended beyond AD, to include decolonization of *S. aureus* to diminish the risk for patients on dialysis to develop catheter‐related infections or prevent infectious complications post‐surgery.

Limitations of this study include the short timeframe of assessment and hence future studies could include an additional 14, 28 or 42 days follow‐up readout to better understand the stability of the treatment effects, both in regards to microbiological but also clinical symptoms endpoint.

Interestingly, ATx201 achieves similar outcomes in AD patients as is expected from several treatments using live bacterial preparations. However, in contrast to such microbial therapies ATx201 would likely offer more effective manufacturing and reduce safety concerns related to bacterial products or donor derived materials.

## CONFLICT OF INTEREST

E.D., A.W., C.M., D.S., P.L. and J.P. were all employees of UNION therapeutics during conduct of study. R.T.K., A.W. and M.O.A.S. are shareholders of UNION therapeutics or have options in UNION therapeutics. E.D., D.S., M.O.A.S., R.T.K. are inventors of patent WO2016038035A1. E.G. and G.S. are advisors for UNION therapeutics. All other authors declare no competing interests.

## Supporting information

SUPPORTING INFORMATION
