# Supplementary material for: Topical niclosamide (ATx201) reduces Staphylococcus aureus colonization and increases Shannon diversity of the skin microbiome in atopic dermatitis patients in a randomized, double‐blind, placebo‐controlled Phase 2 trial
Source: Clin Transl Med. 2022 May 6;12(5):e790. doi: 10.1002/ctm2.790 (PMC9076020; doi:10.1002/ctm2.790)
Supplement: Supplementary file 1 — SUPPORTING INFORMATION [file CTM2-12-e790-s001.docx]

**Supplementary Information**

**Topical Niclosamide (ATx201) Reduces *Staphylococcus aureus* Colonization and Increases Shannon Diversity of The Skin Microbiome in Atopic Dermatitis Patients in a Randomized, Double-blind, Placebo-controlled Phase 2 Trial**

Anne Weiss^1,16,∗^, Emilie Delavenne^1,∗^, Carina Matias^1,∗^, Heimo Lagler^2,∗^, Daniel Simon^1,#^, Ping Li^1^, Jon U. Hansen^3^, Teresa Pires dos Santos^4^, Bimal Jana^4^, Petra Priemel^5^, Christine Bangert^6^, Martin Bauer^7^, Sabine Eberl^7^, Alina Nussbaumer-Pröll^7^, Zoe Anne Österreicher^7^, Peter Matzneller^7^, Tamara Quint^6^, Maria Weber^7^, Hanne Mørck Nielsen^5^, Thomas Rades^5^, Helle Krogh Johansen^8,9^, Henrik Westh^10^, Wooseong Kim^11^, Eleftherios Mylonakis^11^, Christian Friis^4^, Luca Guardabassi^4^, John Pace^1^, Carina Vingsbo Lundberg^3^, Fatima M’Zali^12^, Pascal Butty^13^, Nikolaj Sørensen^14^, Henrik Bjørn Nielsen^14^, Rasmus Toft-Kehler^1^, Emma Guttman-Yassky^15^, Georg Stingl^6^, Markus Zeitlinger^7^, Morten Sommer^1,16^

^1^UNION therapeutics, Hellerup, Denmark

^2^Department of Medicine 1, Division of Infectious Diseases and Tropical Medicine, Medical University of Vienna, Wien, Austria

^3^Department of Bacteria, Parasites and Fungi, Statens Serum Institut, Copenhagen, Denmark ^4^Department of Veterinary and Animal Sciences, University of Copenhagen, Frederiksberg C, Denmark

^5^Department of Pharmacy, University of Copenhagen, Copenhagen, Denmark

^6^Department of Dermatology, Medical University of Vienna, Wien, Austria

^7^Department of Clinical Pharmacology, Medical University of Vienna, Wien, Austria

^8^Department of Clinical Microbiology, Rigshospitalet, Copenhagen, Denmark

^9^Department of Clinical Medicine, Faculty of Health and Medical Sciences, University of Copenhagen, Copenhagen, Denmark

^10^Hvidovre Hospital, Hvidovre, Denmark

^11^Warren Alpert Medical School of Brown University, Division of Infectious Diseases, Rhode Island Hospital, Providence, Rhode Island, USA

^12^University of Bordeaux, Bordeaux Cede

^13^CEVA Santé Animale, Libourne, France

^14^Clinical Microbiomics, Copenhagen, Denmark

^15^Icahn School of Medicine at Mount Sinai, New York, New York, USA

^16^Novo Nordisk Foundation for Biosustainability, Technical University of Denmark, Lyngby, Denmark

^*^ Shared first authorship. These authors contributed equally to this work.

Correspondence should be directed to M.O.A.S, PhD: [morten.sommer@uniontherapeutics.com](mailto:morten.sommer@uniontherapeutics.com), Tuborg Havnevej 18, 2900 Hellerup, Denmark, and M.Z., PhD: [markus.zeitlinger@meduniwien.ac.at](mailto:markus.zeitlinger@meduniwien.ac.at), Department of Clinical Pharmacology, Medical University of Vienna, Spitalgasse 23, 1090 Wien, Austria.

^#^ current address: Faculty of Engineering and Computer Science, NYU Shanghai, 155 Century Avenue, Shanghai 20012, China

Short title: ATx201 as decolonizing agent in atopic dermatitis

**Table of Content**

[Material and Methods 5](#_Toc97554107)

[Figure S1: 1× MIC of standard DNA, RNA, protein and cell wall synthesis inhibitor do not arrest growth of exponentially grown ATCC29213. 8](#_Toc97554108)

[Figure S2: ATx201 acts as a proton carrier. 9](#_Toc97554109)

[Figure S3: Study design of the placebo-controlled, double-blind, split-body-designed Phase 2B study. 10](#_Toc97554110)

[Figure S4: Boxplot of modified EASI and lesional VAS score per treatment regimen 11](#_Toc97554111)

[Figure S5: Genus-level relative abundances per treatment regimen. Dotted line visualizes each paired sample of the same subject. 12](#_Toc97554112)

[Figure S6: Genus-level relative abundance per treatment regimen as stacked plot 15](#_Toc97554113)

[Figure S7: Genus-level relative abundance per patient as stacked plot, separated in the once and twice daily treatment group 16](#_Toc97554114)

[Figure S7: Genus-level relative abundance per patient as stacked plot, separated in the once and twice daily treatment group - continued 17](#_Toc97554115)

[Table S1: CONSORT 2010 checklist of information to include when reporting a randomised trial 18](#_Toc97554116)

[Table S2: Full list of inclusion and exclusion criteria 21](#_Toc97554117)

[Table S3: Visit and assessment schedule 23](#_Toc97554118)

[Table S4: Definitions used to grade the relationship of an adverse event to study drug 24](#_Toc97554119)

[Table S5: *in vitro* susceptibility of *S. aureus* clinical isolates and *S. aureus* ATCC 29213 reference strain. 25](#_Toc97554120)

[Table S6: Mutation rates (µ) and frequencies of resistant mutants (*F*) for rifampicin in *S. aureus* strains 36](#_Toc97554121)

[Table S7: Baseline demographics per regimen group 37](#_Toc97554122)

[Table S8: Summary of AEs by Organ class, relationship to treatment, outcome, and detailed description of administration site conditions. The split-body design of the clinical trial precludes assessing systemic AEs per treatment regimen. 38](#_Toc97554123)

[Table S9: Summary of modified EASI, lesional VAS and lesional IGA score 42](#_Toc97554124)

[Table S10: MICs of ATx201 in culturable strains isolated from patients at Day 7 43](#_Toc97554125)

# Material and Methods

*Quantification of skin microbiome diversity*

DNA was extracted from skin swaps using NucleoSpin® 96 Soil (Macherey-Nagel). rRNA genes (16S) were amplified from purified genomic DNA using primers 515F (5'- CGTCGGCAGCGTCAGATGTGTATAAGAGACAGGTGYCAGCMGCCGCGGTAA-3') and 806R (5'-GTCTCGTGGGCTCGGAGATGTGTATAAGAGACAGGGACTACNVGGGTWTCTAAT-3’) with Ilumina adapter attached. Sequencing was done on an Illumina MiSeq desktop sequencer using the MiSeq Reagent Kit V3 (Illumina) for 2x 300 bp paired-end sequencing. The 64-bit version of USEARCH 10.0 (1), mothur 1.38 (2), and in-house scripts were used for bioinformatics analysis of the sequence data. Taxonomic assignment of OTUs was done using SINTAX with a cutoff value of 0.8 (3) against RDP training set v16 (4). All analyses, except for calculation of Shannon index, were done on rarefied data. The α-diversity of the samples was assessed by counting the number of OTUs (operational taxonomical units, similar to species richness) and calculating the Shannon index. The Shannon index does not only take the number of OTUs of a community into account, but also the relative abundance of the OTUs. It will give a low diversity score to a community dominated by a few species (even though the total species number may be high), while communities where many different species have similar abundances will receive a high diversity score.

The β-diversity is a measure of dissimilarity in the taxonomic composition between samples. One measure of β-diversity is UniFrac. The UniFrac distance takes taxonomic distance of the OTUs into account: two samples consisting of different species from the same genus will have a shorter UniFrac distance between them than two samples consisting of species from different phyla. The UniFrac distances can also take abundance into account. If full weight is given to the abundances (α = 1, where α determines the weight given to abundance differences) it is called a weighted UniFrac. In such case, the UniFrac distance may be high between two samples that contain the exact same species in very different abundances. A UniFrac distance that does not take abundance into account (α = 0), working only with presence-absence data, is called an unweighted UniFrac.

**References**

1. Edgar RC. UPARSE: highly accurate OTU sequences from microbial amplicon reads. Nat Methods. 2013;

2. Schloss PD, Westcott SL, Ryabin T, Hall JR, Hartmann M, Hollister EB, et al. Introducing mothur: Open-source, platform-independent, community-supported software for describing and comparing microbial communities. Appl Environ Microbiol. 2009;

3. Edgar R. SINTAX: a simple non-Bayesian taxonomy classifier for 16S and ITS sequences. bioRxiv. 2016;074161.

4. Cole JR, Wang Q, Fish JA, Chai B, McGarrell DM, Sun Y, et al. Ribosomal Database Project: Data and tools for high throughput rRNA analysis. Nucleic Acids Res. 2014;

# Figure S1: 1× MIC of standard DNA, RNA, protein and cell wall synthesis inhibitor do not arrest growth of exponentially grown ATCC29213.

Cells were grown to 0.3 OD at 600 nm then distributed to separate tubes and incubated with 1× and 4× and 10× MIC of ciprofloxacin, rifampicin, erythromycin, vancomycin or ATx201 for another 3 hours and cell OD was recorded after every hour and plotted in growth curve. In the right panel, MIC fold of antimicrobial concentrations are listed, which follow the same order as growth curve lines from top to bottom. 1× MIC of ciprofloxacin, rifampicin, erythromycin, vancomycin and ATx201 are 0.25, 0.003, 0.5, 2 and 0.25 µg/mL, respectively.

# Figure S2: ATx201 acts as a proton carrier.

Effect of ATx201, nigericin, CCCP or DMSO treatments on BCECF fluorescence in ATCC 29213 are plotted as function of time.


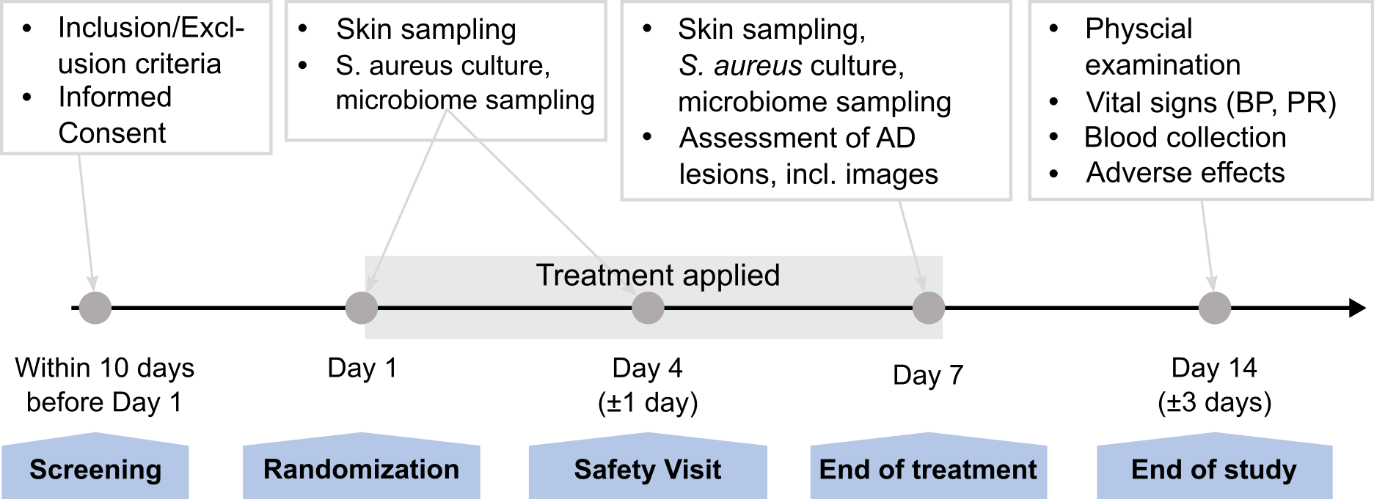


# Figure S3: Study design of the placebo-controlled, double-blind, split-body-designed Phase 2B study.

**
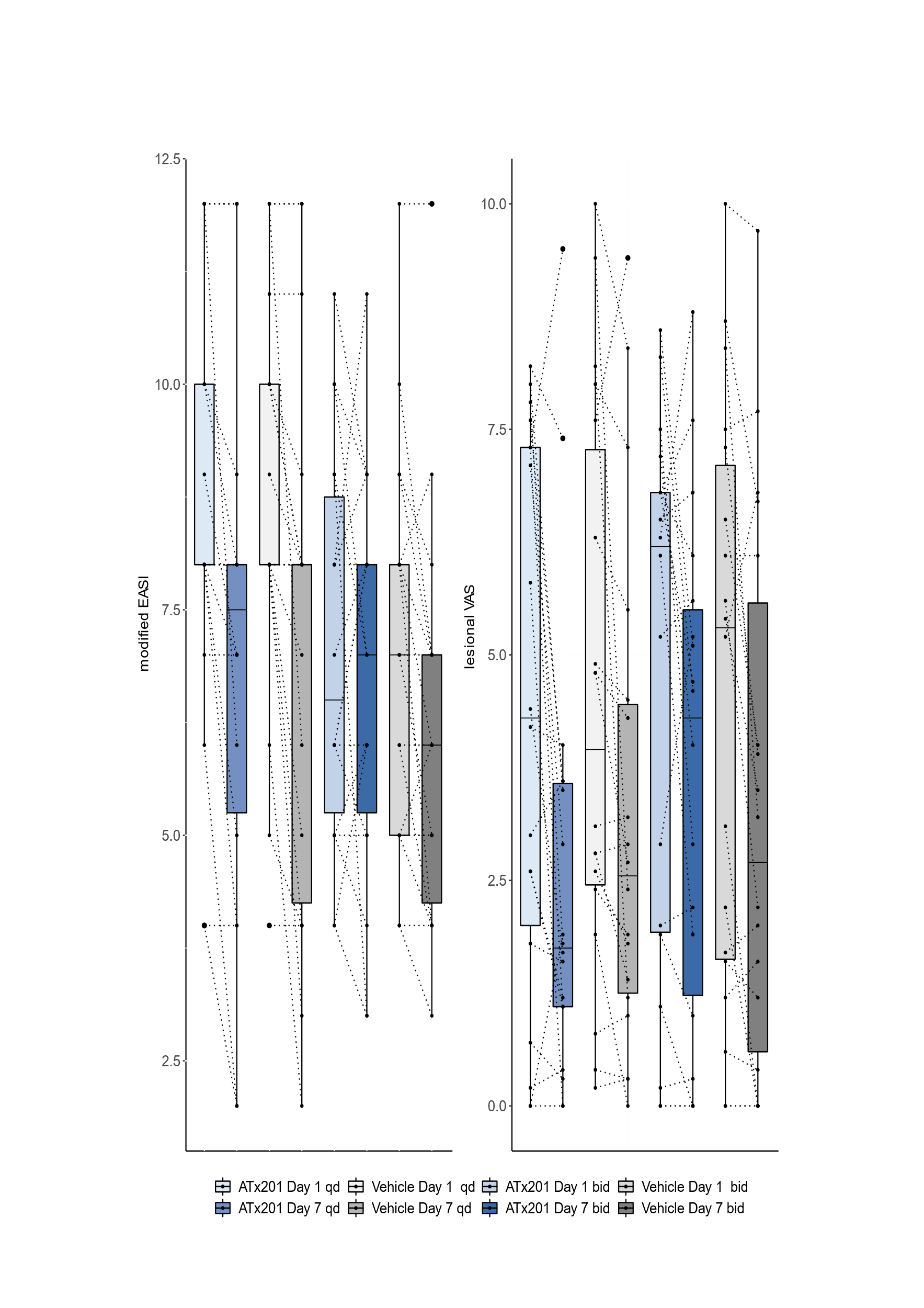
**

# Figure S4: Boxplot of modified EASI and lesional VAS score per treatment regimen


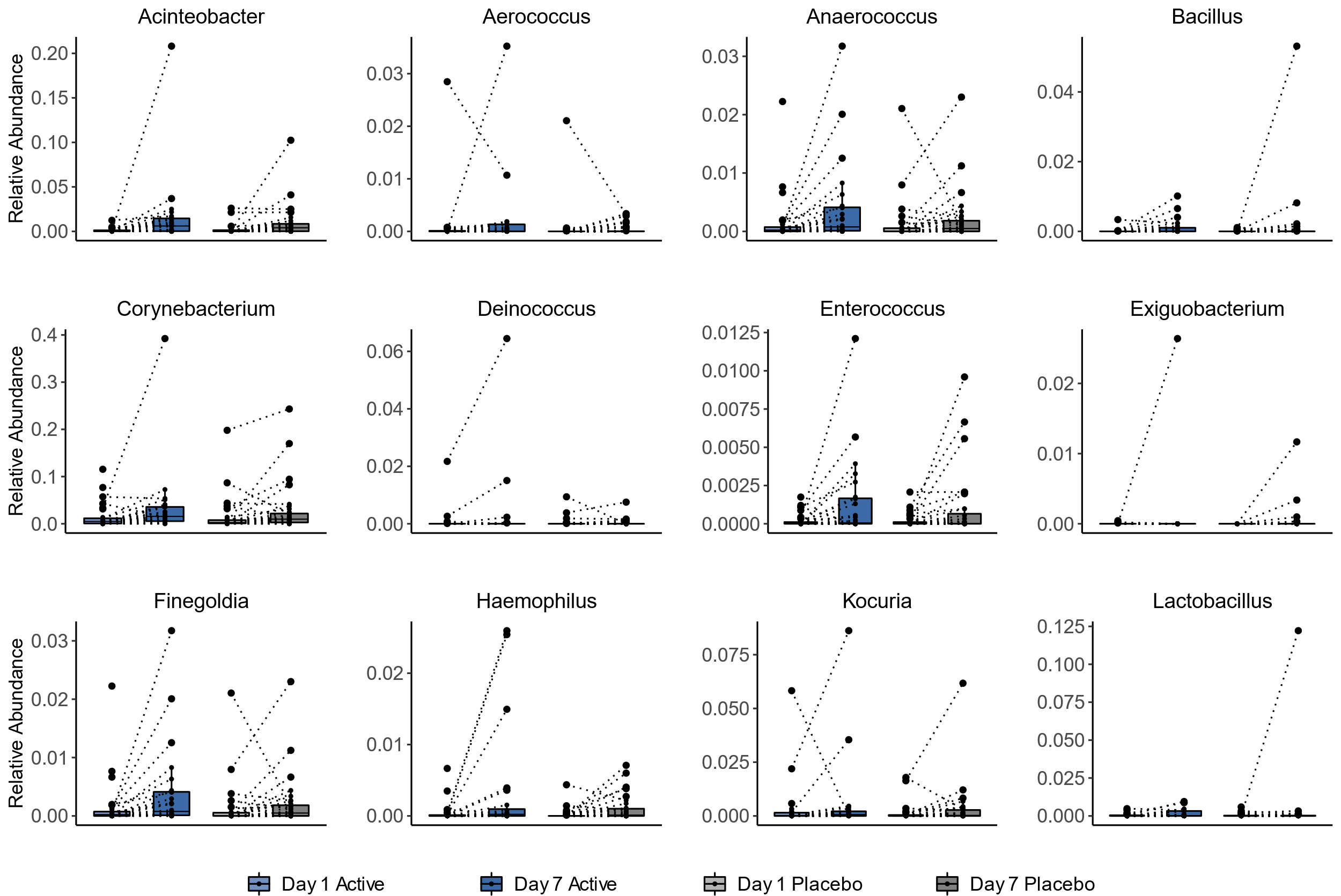


# Figure S5: Genus-level relative abundances per treatment regimen. Dotted line visualizes each paired sample of the same subject.


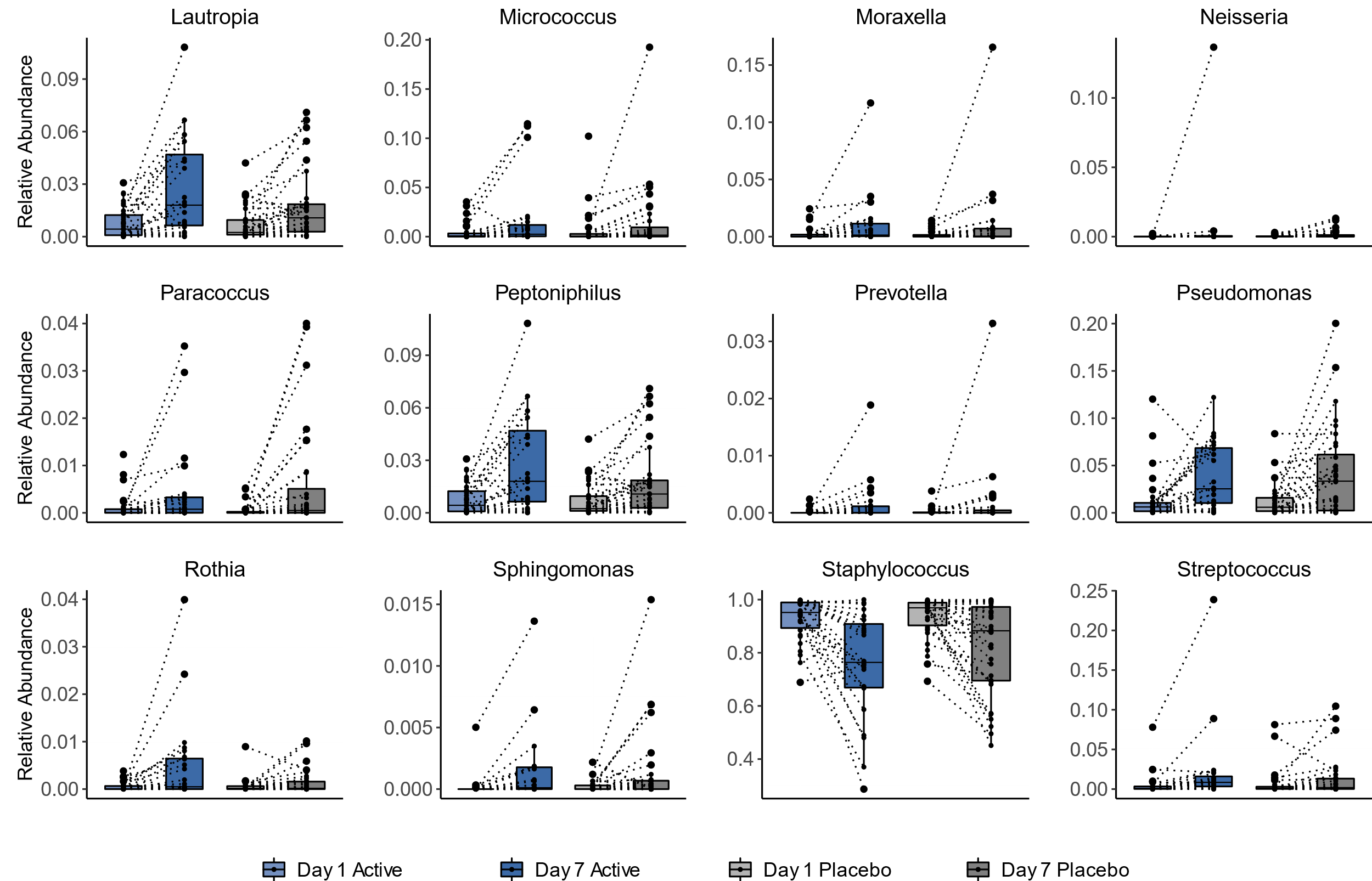


**Figure S5: Genus-level relative abundances per treatment regimen – continued.**


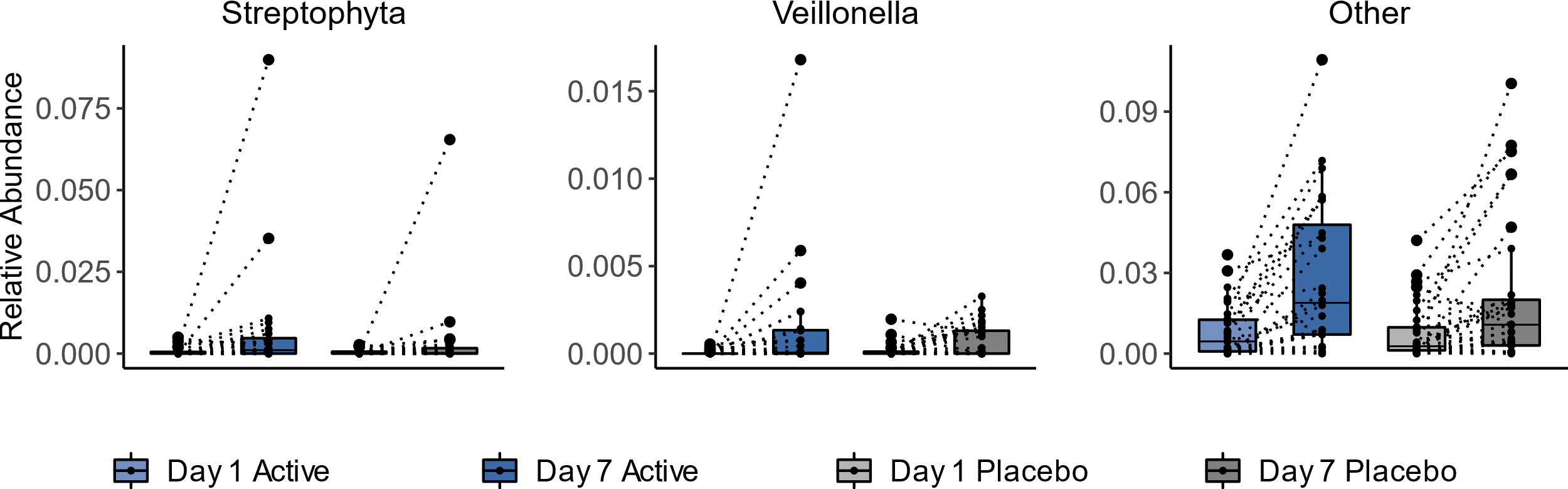


**Figure S5: Genus-level relative abundances per treatment regimen – continued.**


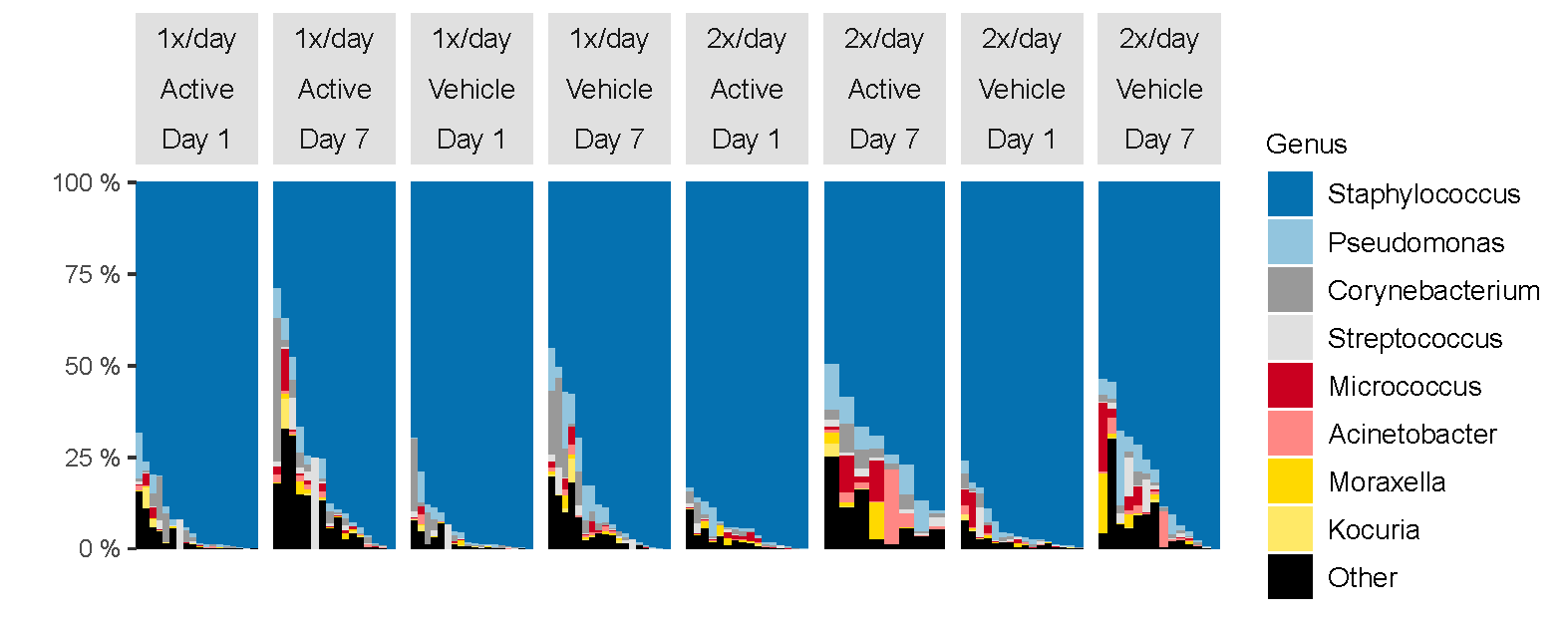


# Figure S6: Genus-level relative abundance per treatment regimen as stacked plot

# Figure S7: Genus-level relative abundance per patient as stacked plot, separated in the once and twice daily treatment group

# Figure S7: Genus-level relative abundance per patient as stacked plot, separated in the once and twice daily treatment group - continued

# Table S1: CONSORT 2010 checklist of information to include when reporting a randomised trial

| Section/Topic | Item No | Checklist item | Reported on page No |
| --- | --- | --- | --- |
| Title and abstract | | | |
|  | 1a | Identification as a randomised trial in the title | 1 |
|  | 1b | Structured summary of trial design, methods, results, and conclusions (for specific guidance see CONSORT for abstracts) | 3 |
| Introduction | | | |
| Background and objectives | 2a | Scientific background and explanation of rationale | 4-6 |
|  | 2b | Specific objectives or hypotheses | 6 |
| Methods | | | |
| Trial design | 3a | Description of trial design (such as parallel, factorial) including allocation ratio | 13 |
|  | 3b | Important changes to methods after trial commencement (such as eligibility criteria), with reasons | NA |
| Participants | 4a | Eligibility criteria for participants | 13 + supporting information |
|  | 4b | Settings and locations where the data were collected | 13 |
| Interventions | 5 | The interventions for each group with sufficient details to allow replication, including how and when they were actually administered | 13-14 |
| Outcomes | 6a | Completely defined pre-specified primary and secondary outcome measures, including how and when they were assessed | 14-15 |
|  | 6b | Any changes to trial outcomes after the trial commenced, with reasons | NA |
| Sample size | 7a | How sample size was determined | 17-18 |
|  | 7b | When applicable, explanation of any interim analyses and stopping guidelines | NA |
| Randomisation: |  |  |  |
| Sequence generation | 8a | Method used to generate the random allocation sequence | 15 |
|  | 8b | Type of randomisation; details of any restriction (such as blocking and block size) | 15 |
| Allocation concealment mechanism | 9 | Mechanism used to implement the random allocation sequence (such as sequentially numbered containers), describing any steps taken to conceal the sequence until interventions were assigned | 15 |
| Implementation | 10 | Who generated the random allocation sequence, who enrolled participants, and who assigned participants to interventions | 15 |
| Blinding | 11a | If done, who was blinded after assignment to interventions (for example, participants, care providers, those assessing outcomes) and how | 15 |
|  | 11b | If relevant, description of the similarity of interventions | 16 |
| Statistical methods | 12a | Statistical methods used to compare groups for primary and secondary outcomes | 17-18 |
|  | 12b | Methods for additional analyses, such as subgroup analyses and adjusted analyses | NA |
| Results | | | |
| Participant flow (a diagram is strongly recommended) | 13a | For each group, the numbers of participants who were randomly assigned, received intended treatment, and were analysed for the primary outcome | 24 |
|  | 13b | For each group, losses and exclusions after randomisation, together with reasons | 24 |
| Recruitment | 14a | Dates defining the periods of recruitment and follow-up | NA |
|  | 14b | Why the trial ended or was stopped | NA |
| Baseline data | 15 | A table showing baseline demographic and clinical characteristics for each group | 25 (suppl. Info.) |
| Numbers analysed | 16 | For each group, number of participants (denominator) included in each analysis and whether the analysis was by original assigned groups | 24 |
| Outcomes and estimation | 17a | For each primary and secondary outcome, results for each group, and the estimated effect size and its precision (such as 95% confidence interval) | 25-27 |
|  | 17b | For binary outcomes, presentation of both absolute and relative effect sizes is recommended | NA |
| Ancillary analyses | 18 | Results of any other analyses performed, including subgroup analyses and adjusted analyses, distinguishing pre-specified from exploratory | NA |
| Harms | 19 | All important harms or unintended effects in each group (for specific guidance see CONSORT for harms) | NA |
| Discussion | | | |
| Limitations | 20 | Trial limitations, addressing sources of potential bias, imprecision, and, if relevant, multiplicity of analyses | 31 |
| Generalisability | 21 | Generalisability (external validity, applicability) of the trial findings | 30-31 |
| Interpretation | 22 | Interpretation consistent with results, balancing benefits and harms, and considering other relevant evidence | 30-31 |
| Other information | | |  |
| Registration | 23 | Registration number and name of trial registry | 13 |
| Protocol | 24 | Where the full trial protocol can be accessed, if available | Supporting information |
| Funding | 25 | Sources of funding and other support (such as supply of drugs), role of funders | 34 |

# Table S2: Full list of inclusion and exclusion criteria

| **Inclusion criteria** |
| --- |
| 1. Signed and dated informed consent has been obtained  2. Age 18-70 years  3. Male or female  4. Female subjects of childbearing potential had to be confirmed not pregnant by a negative urine pregnancy test prior trial treatment  5. Female subjects of childbearing potential had to be willing to use effective contraceptive at trial entry until completion  6. Male subjects had to agree to use adequate contraception for the duration of the trial  7. Localized disease where two individual lesions each covering an area between 10-200 cm2 and where each individual lesion has a lesional IGA score between 1-4 and is colonized by *S. aureus* with at least 1,000 CFU/cm2.  8. Additional localized lesion of area between 10-200 cm2 and where the individual lesion has a lesional IGA score between 1 and 4. |
| **Exclusion criteria** |
| 1. Clinically relevant abnormalities in the laboratory testing, vital signs, ECG (Part 1 only) or physical examination unless considered clinically irrelevant for the scope of the trial by the investigator.  2. Presence of any skin condition (scars, tattoos, etc.) that would interfere with the placement of study medication.  3. History of irritation to topical products.  4. Current acute or chronic disease unless considered clinically irrelevant for the scope of the trial by the investigator.  5. Relevant history of malignancy, of renal, hepatic, cardiovascular, respiratory, gastrointestinal, musculoskeletal, skin (particularly at the site of drug application), hematological, endocrine or neurological diseases that may interfere with the aim of the study.  6. Positive HIV serology or evidence of active hepatitis.  7. Ascertained or presumptive hypersensitivity to the active principle and/or formulations ingredients of the study drugs (test, reference).  8. History of drug or alcohol abuse (>2 drinks/day, defined according to USDA Dietary Guidelines 2005).  9. Blood donations during 6 weeks prior to this study or planned within 6 weeks after the last blood withdrawal.  10. Subject considered unable or unlikely (per investigator judgement) to comply with safety and PK profiling requirements (follow-up visits).  11. Subjects who are pregnant (as determined by a positive pregnancy test at the screening Visit) or lactating.  12. Participation in another clinical trial with an investigational drug within 4 weeks before screening.  13.Treatment with antibiotics (systemic or topical) within the past 2 months taken for systemic application, and 4 weeks use of topical antibiotics prior entering the study, and use of those during the study.  14. Treatment with cyclosporins within the last four weeks, use of methotrexate or mycophenolate mofertil within the last eight weeks, and biologicals within 5 times the biological half-life prior to entering the study.  15. Treatment with topical (dermatological) steroids and calcineurin inhibitors 1 week prior to start of treatment and during the study.  16. Treatment with systemic steroids within the past month and during the study.  17. Use of disinfectant soaps within 1 week before screening and during the study treatment period. |

# Table S3: Visit and assessment schedule

**
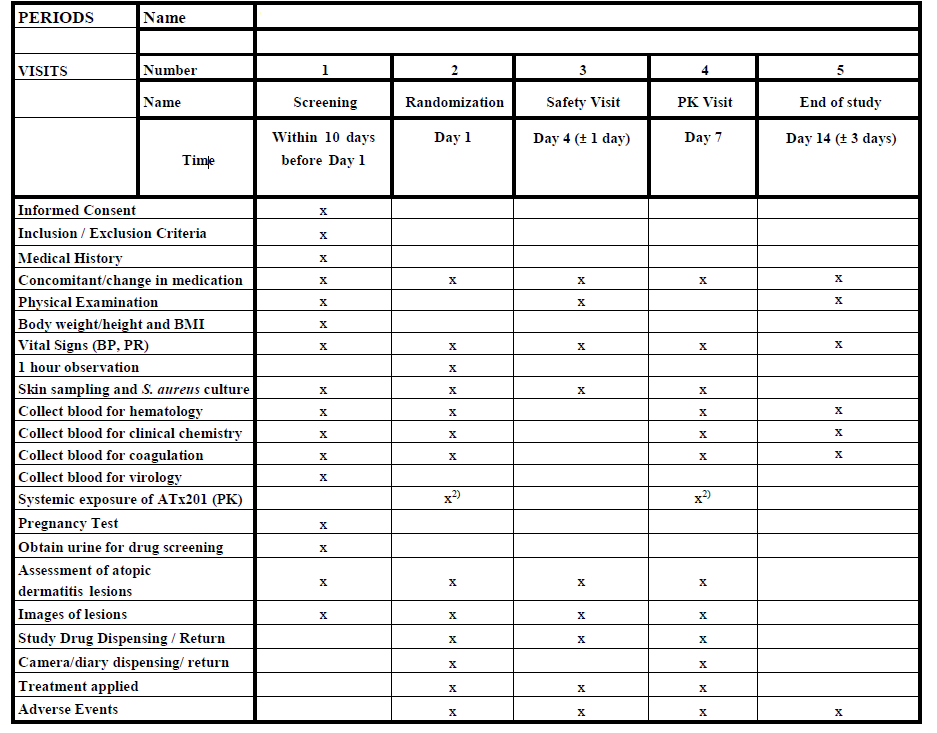
**

1) Patients in the twice daily treated group received their last dose in the morning on Day 7.

2) One blood sample was taken for pharmacokinetic assessment at Day 7 from 30 patients. Ten patients (5 treated once-daily and 5 treated twice-daily) entered an extended pharmacokinetic assessment with one blood sample taken prior to first application at Day 1. On Day 7 these patients had blood samples taken within one hour before last application and 1, 2, 4, 6 and 8 hours after the last application.

# Table S4: Definitions used to grade the relationship of an adverse event to study drug

| For all adverse events (AEs), the Investigator will assess the causal relationship between the study drug and the AE using his/her clinical expertise and judgment according to the following algorithm that best fits the circumstances of the AE: | |
| --- | --- |
| **Not Related** | - May or may not follow a temporal sequence from administration of the study product - Is biologically implausible and does not follow known response pattern to the suspect study drug (if response pattern is previously known). - Can be explained by the known characteristics of the subject’s clinical state or other modes of therapy administered to the subject. |
| **Unlikely** | - There is a reasonable temporal relation between the AE and the intake of the study medication, but there is a plausible other explanation for the occurrence of the AE |
| **Possibly** | - The AE has a reasonable temporal relationship with drug administration. - The AE may equally be explained by the study subject’s clinical state, environmental or toxic factors, or concomitant therapy administered to the study subject. - The relationship between study drug and AE may also be pharmacologically or clinically plausible. |
| **Probably** | - There is a reasonable temporal relation between the AE and the intake of the study medication, and plausible reasons point to a causal relation with the study medication. |
| **Related** | - Reasonable temporal relation between the AE and the intake of the study medication and - there is no other explanation for the AE and - subsidence or disappearance of the AE on withdrawal of the study medication and - recurrence of the symptoms on restart at previous dose (only applies for re-institution of mediation). |
| **Not assessable** | - The causal relationship between the study drug and the AE can not be judged. |

AE = adverse event

# Table S5: *in vitro* susceptibility of *S. aureus* clinical isolates and *S. aureus* ATCC 29213 reference strain.

Values above clinical breakpoint are highlighted in red. ND: not determined

|  | **MIC (µg/mL)** | | | | | |
| --- | --- | --- | --- | --- | --- | --- |
|  | **ATx201** | **Fusidic acid** | **Mupirocin** | **Retapamulin** | **Clindamycin** | **Vancomycin** |
| ATCC 29213 | 0.5 | 0.06 | 0.125 | 0.03 | 0.125 | 1 |
| Newman | 0.25 | 0.25 | 0.25 | 0.03 | 0.125 | 2 |
| MRSA 01 | 0.25 | 0.125 | 0.25 | 0.06 | 0.125 | 1 |
| MRSA 02 | 0.25 | 0.5 | 0.25 | 0.06 | 0.25 | 2 |
| MRSA 03 | 0.25 | 16 | 0.25 | 0.03 | 0.125 | 1 |
| MRSA 04 | 0.25 | 0.25 | 0.25 | 0.06 | 0.125 | 2 |
| MRSA 05 | 0.25 | 0.125 | 0.125 | 0.03 | 0.125 | 1 |
| MRSA 06 | 0.25 | 0.125 | 0.25 | 0.03 | 0.125 | 2 |
| MRSA 07 | 0.125 | 0.25 | 0.25 | 0.06 | 0.125 | 1 |
| MRSA 08 | 0.25 | 0.125 | > 16 | 0.03 | > 16 | 1 |
| MRSA 09 | 0.125 | 0.25 | 0.25 | 0.06 | 0.25 | 2 |
| MRSA 10 | 0.25 | 1 | 0.25 | 0.06 | 0.25 | 1 |
| MRSA 11 | 0.25 | 1 | 0.5 | 0.06 | > 16 | 1 |
| EEFIC 01 | 0.25 | 4 | 0.125 | 0.03 | 0.125 | 1 |
| EEFIC 02 | 0.25 | 4 | 0.125 | 0.03 | 0.125 | 1 |
| MRSA 12 | 0.5 | 4 | 0.125 | 0.03 | ND | ND |
| MRSA 13 | 0.25 | 4 | 0.25 | 0.06 | 0.125 | 1 |
| MSSA 01 | 0.5 | 4 | 0.125 | 0.03 | 0.125 | 1 |
| MSSA 02 | 0.25 | 4 | 0.125 | 0.03 | 0.125 | 1 |
| MRSA 14 | 0.25 | > 16 | 0.125 | 0.03 | > 16 | 1 |
| MRSA 15 | 0.25 | > 16 | 0.25 | 0.06 | 0.125 | 2 |
| MRSA 16 | 0.25 | 0.25 | > 16 | 0.06 | 0.25 | 1 |
| MRSA 17 | 0.25 | 4 | > 16 | 0.03 | > 16 | 2 |
| MRSA 18 | 0.25 | 0.25 | 0.125 | 0.06 | 0.125 | 1 |
| MRSA 19 | 0.25 | 0.125 | 0.25 | 0.03 | 0.125 | 1 |
| MRSA 20 | 0.25 | 0.125 | 0.25 | 0.06 | 0.06 | 1 |
| MRSA 21 | 0.25 | 0.125 | 0.125 | 0.125 | 0.03 | 1 |
| MRSA 22 | 0.25 | > 16 | 0.25 | 0.06 | 0.06 | 0.5 |
| MRSA 23 | 0.25 | 0.25 | 0.25 | 0.06 | 0.125 | 1 |
| MRSA 24 | 0.25 | 0.25 | 0.25 | 0.06 | 0.125 | 2 |
| MRSA 25 | 0.25 | 16 | 0.25 | 0.06 | 0.125 | 1 |
| MRSA 26 | 0.5 | 0.5 | 0.5 | 0.06 | > 16 | 1 |
| MRSA 27 | 0.5 | 0.25 | 0.25 | 0.06 | 0.125 | 1 |
| MRSA 28 | 0.25 | 0.06 | 0.5 | 0.03 | 0.06 | 1 |
| MRSA 29 | 0.5 | 4 | 0.25 | 0.06 | 0.125 | 1 |
| MRSA 30 | 0.25 | 0.125 | 0.125 | 0.03 | 0.06 | 1 |
| MRSA 31 | 0.25 | 0.25 | 0.25 | 16 | > 16 | 1 |
| MRSA 32 | 0.25 | 0.5 | 0.25 | 0.06 | 0.125 | 1 |
| MRSA 33 | 0.25 | 0.5 | 0.25 | 0.06 | 0.125 | 1 |
| MRSA 34 | 0.25 | 0.25 | 0.25 | 0.06 | 0.125 | 1 |
| MRSA 35 | 0.25 | 16 | 0.5 | 0.03 | > 16 | 1 |
| MRSA 36 | 0.25 | 8 | 0.25 | 0.06 | 0.06 | 1 |
| MRSA 37 | 0.25 | 0.5 | 0.25 | 0.06 | 0.125 | 1 |
| MRSA 38 | 0.25 | 8 | 0.125 | 0.06 | 0.125 | 1 |
| MRSA 39 | 0.25 | 8 | 0.25 | 0.06 | 0.06 | 1 |
| MRSA 40 | 0.25 | 0.125 | 0.25 | 0.03 | 0.06 | 1 |
| MRSA 41 | 0.25 | > 16 | 0.125 | 0.02 | 0.06 | 1 |
| MRSA 42 | 0.25 | 0.25 | 0.25 | 0.06 | > 16 | 1 |
| MRSA 43 | 0.25 | 0.125 | 0.25 | 0.06 | 0.06 | 1 |
| MRSA 44 | 0.25 | 4 | 0.25 | 0.02 | 0.03 | 1 |
| MRSA 45 | 0.25 | 0.06 | 0.13 | 0.02 | 0.06 | 1 |
| MRSA 46 | 0.25 | 0.06 | 0.13 | 0.02 | 0.03 | 1 |
| MRSA 47 | 0.25 | 0.06 | 0.25 | 0.02 | > 16 | 0.5 |
| MRSA 48 | 0.25 | 2 | 0.13 | 0.02 | 0.03 | 0.5 |
| MRSA 49 | 0.25 | 0.25 | 0.25 | 0.02 | 0.03 | 2 |
| MRSA 50 | 0.25 | 0.03 | 0.25 | 0.02 | 0.02 | 1 |
| MRSA 51 | 0.125 | 0.13 | 0.25 | 0.03 | 0.06 | 1 |
| MRSA 52 | 0.25 | 0.06 | 0.25 | 0.02 | 0.03 | 1 |
| MRSA 53 | 0.25 | 0.25 | 0.25 | 0.03 | 0.06 | 1 |
| MRSA 54 | 0.25 | 0.125 | 0.25 | 0.03 | 0.06 | 2 |
| MRSA 55 | 0.25 | 4 | 0.25 | 0.02 | 0.03 | 0.5 |
| MRSA 56 | 0.25 | 0.06 | 0.25 | < 0.01 | 0.03 | 1 |
| MRSA 57 | 0.25 | 0.125 | 0.125 | 0.02 | 0.03 | 1 |
| MRSA 58 | 0.25 | 4 | 0.125 | 0.02 | 0.06 | 1 |
| MRSA 59 | 0.25 | 0.06 | 0.125 | 0.02 | 0.03 | 1 |
| MRSA 60 | 0.25 | 8 | 0.25 | 0.02 | 0.06 | 0.5 |
| MRSA 61 | 0.25 | 0.06 | 0.25 | 0.02 | 0.03 | 2 |
| MRSA 62 | 0.25 | 0.06 | 0.25 | 0.02 | 0.06 | 2 |
| MRSA 63 | 0.25 | 0.125 | 0.125 | 0.02 | 0.06 | 1 |
| MRSA 64 | 0.25 | 0.25 | 0.25 | 0.02 | 0.06 | 1 |
| MRSA 65 | 0.25 | 0.06 | 0.25 | 0.02 | 0.03 | 1 |
| MRSA 66 | 0.25 | 0.06 | 0.25 | 0.02 | 0.03 | 1 |
| MRSA 67 | 0.25 | 0.25 | 0.125 | 0.02 | 0.03 | 1 |
| MRSA 68 | 0.25 | 0.125 | 0.25 | 0.02 | 0.03 | 1 |
| MRSA 69 | 0.25 | 4 | 0.125 | < 0.01 | 0.03 | 1 |
| MRSA 70 | 0.125 | 8 | 0.25 | 0.02 | 0.06 | 1 |
| MRSA 71 | 0.25 | 0.06 | 0.125 | < 0.01 | 0.02 | 1 |
| MRSA 72 | 0.25 | 16 | 0.25 | 0.03 | 0.06 | 1 |
| MRSA 73 | 0.5 | 0.25 | 0.25 | 0.03 | 0.125 | 1 |
| MRSA 74 | 0.25 | 0.25 | 0.25 | 0.03 | 0.125 | 2 |
| MRSA 75 | 0.5 | 0.5 | 0.5 | 0.03 | 0.125 | 1 |
| MRSA 76 | 0.25 | 0.25 | 0.25 | 0.03 | 0.125 | 1 |
| MRSA 77 | 0.25 | 16 | 0.25 | 0.03 | 0.125 | 2 |
| MRSA 78 | 0.5 | 0.125 | 0.25 | 0.03 | 0.06 | 1 |
| MRSA 79 | 0.25 | 0.5 | 0.25 | < 0.01 | 0.03 | 1 |
| MRSA 80 | 0.5 | 0.125 | 0.25 | 0.02 | 0.06 | 1 |
| MRSA 81 | 0.5 | 0.25 | 0.25 | 0.03 | 0.125 | 1 |
| MRSA 82 | 0.25 | 8 | 0.25 | 0.03 | 0.125 | 2 |
| MRSA 83 | 0.5 | 0.06 | 0.25 | 0.03 | 0.06 | 1 |
| MRSA 27b | 0.25 | 8 | 0.25 | 0.02 | 0.06 | 1 |
| MRSA 84 | 0.25 | 8 | 0.25 | 0.03 | 0.125 | 2 |
| MRSA 85 | 0.5 | 4 | 0.25 | 0.03 | 0.125 | 1 |
| MRSA 86 | 0.25 | > 16 | 0.25 | 0.03 | 0.06 | 2 |
| MRSA 87 | 0.25 | 0.125 | 0.25 | 0.02 | 0.06 | 1 |
| MRSA 88 | 0.25 | 0.06 | 0.25 | 0.03 | 0.06 | 1 |
| MRSA 89 | 0.5 | 0.06 | 0.25 | 0.02 | > 16 | 1 |
| MRSA 90 | 0.5 | 16 | 0.25 | 0.06 | 0.125 | 1 |
| MRSA 91 | 0.25 | 0.25 | 0.25 | 0.06 | 0.125 | 1 |
| MRSA 92 | 0.25 | 8 | 0.25 | 0.06 | 0.06 | 1 |
| MRSA 93 | 0.25 | 0.02 | 0.25 | 0.02 | 0.03 | 2 |
| MRSA 94 | 0.25 | 0.125 | 0.25 | 0.03 | 0.125 | 2 |
| MRSA 95 | 0.25 | 8 | 0.25 | 0.03 | 0.125 | 2 |
| MRSA 96 | 0.25 | 0.125 | 0.125 | 0.03 | > 16 | 1 |
| MRSA 97 | 0.25 | 8 | 0.25 | 0.03 | 0.06 | 1 |
| MRSA 98 | 0.5 | 0.06 | 0.5 | 0.03 | > 16 | 1 |
| MRSA 99 | 0.25 | 0.125 | 0.5 | 0.03 | 0.125 | 1 |
| MRSA 100 | 0.25 | 0.125 | 0.5 | 1 | 0.25 | 1 |
| MRSA 101 | 0.5 | 8 | 0.25 | 0.03 | 0.06 | 1 |
| MRSA 102 | 0.25 | 0.06 | 0.25 | 0.03 | 0.06 | 2 |
| MRSA 103 | 0.25 | 8 | 0.25 | 0.03 | 0.125 | 1 |
| MRSA 104 | 0.5 | 0.25 | 0.25 | 0.03 | 0.125 | 1 |
| MRSA 105 | 0.25 | 0.125 | 0.25 | 0.03 | 0.125 | 1 |
| MRSA 106 | 0.25 | 0.125 | 0.25 | 0.06 | 0.125 | 1 |
| MRSA 107 | 0.25 | 0.25 | 0.25 | 0.06 | 0.125 | 2 |
| MRSA 108 | 0.25 | 4 | 0.25 | 0.03 | 0.125 | 1 |
| MRSA 109 | 0.25 | 0.25 | 0.25 | 0.03 | 0.125 | 1 |
| MRSA 110 | 0.25 | 0.06 | 0.125 | 0.03 | 0.125 | 1 |
| MRSA 111 | 0.5 | 8 | 0.25 | 0.03 | 0.125 | 1 |
| MRSA 112 | 0.25 | 0.06 | 0.25 | 0.03 | 0.06 | 1 |
| MRSA 113 | 0.25 | 0.125 | 0.25 | 0.03 | 0.06 | 1 |
| K000796 | 0.25 | 8 | 0.5 | 0.03 | 0.125 | 1 |
| K115688 | 0.25 | 0.125 | 0.25 | 0.03 | 0.125 | 2 |
| K000866 | 0.25 | 8 | 0.5 | 0.03 | 0.125 | 1 |
| K000864 | 0.25 | 0.25 | 0.25 | 0.03 | 0.125 | 1 |
| K000863 | 0.25 | 1 | 0.5 | 0.03 | 0.125 | 1 |
| K115689 | 0.25 | 0.125 | 0.25 | 0.03 | 0.125 | 2 |
| K000772 | 0.25 | > 16 | 0.125 | 0.06 | 0.125 | 1 |
| K115498 | 0.25 | 0.125 | 0.5 | 0.03 | 0.125 | 2 |
| R000024 | 0.25 | 16 | 0.5 | 0.06 | 0.125 | 1 |
| R000020 | 0.5 | 0.125 | 0.5 | 0.03 | 0.125 | 1 |
| R000019 | 0.5 | 0.125 | 0.5 | 0.06 | 0.125 | 2 |
| U115579 | 0.25 | 0.25 | 0.5 | 0.06 | 0.125 | 1 |
| U115370 | 0.25 | 0.5 | 0.125 | 0.06 | 0.125 | 1 |
| U114660 | 0.25 | 0.25 | 0.25 | 0.06 | 0.125 | 1 |
| D115584 | 0.25 | 0.25 | 0.5 | 0.06 | 0.125 | 2 |
| E115740 | 0.5 | 0.25 | 0.25 | 0.06 | 0.125 | 1 |
| E115810 | 0.25 | 0.25 | 0.25 | 0.06 | 0.125 | 1 |
| T115628 | 0.25 | 8 | 0.25 | 0.03 | 0.06 | 2 |
| T000274 | 0.25 | 0.5 | 0.5 | 0.06 | 0.125 | 1 |
| T115691 | 0.5 | 0.25 | 0.25 | 0.03 | 0.125 | 1 |
| T115903 | 0.5 | 8 | 0.5 | 0.03 | 0.125 | 1 |
| T116122 | 0.25 | 0.125 | 0.5 | 0.03 | 0.125 | 1 |
| T115015 | 0.5 | 0.25 | 0.5 | 0.06 | 0.125 | 2 |
| C115273 | 0.5 | 0.25 | 0.25 | 0.03 | 0.125 | 1 |
| C000040 | 0.5 | 0.25 | 0.25 | 0.03 | 0.125 | 1 |
| C115690 | 0.25 | 8 | 0.125 | 0.25 | 0.5 | 2 |
| C115561 | 0.25 | 0.125 | 0.5 | 0.03 | 0.125 | 1 |
| C115445 | 0.5 | 0.25 | 0.25 | 0.03 | 0.125 | 1 |
| C115263 | 0.25 | 0.125 | 0.25 | 0.03 | 0.06 | 2 |
| C115303 | 0.5 | 0.25 | 0.25 | 0.03 | > 16 | 1 |
| C115268 | 0.5 | 0.25 | 0.5 | 0.03 | 0.125 | 1 |
| C115295 | 0.25 | 0.125 | 0.25 | 0.03 | 0.125 | 1 |
| C115242 | 0.5 | 8 | 0.25 | 0.03 | 0.06 | 1 |
| C115427 | 0.25 | 0.125 | 0.25 | 0.03 | 0.06 | 1 |
| C000041 | 0.25 | 0.25 | 0.25 | 0.03 | 0.06 | 1 |
| E5-1048654 | 0.25 | 0.5 | 0.25 | 0.06 | 0.125 | 1 |
| 9-2955245 | 0.25 | 0.25 | 0.25 | 0.06 | 0.125 | 1 |
| E5-1046019 | 0.25 | 0.25 | 0.25 | 0.03 | 0.125 | 1 |
| E5-1046020 | 0.5 | 0.25 | 0.25 | 0.06 | 0.125 | 1 |
| E5-1047585 | 0.25 | 0.25 | 0.25 | 0.03 | 0.125 | 1 |
| E5-1038294 | 0.5 | 0.25 | 0.25 | 0.03 | 0.125 | 1 |
| E5-1035779 | 0.5 | 0.125 | 0.5 | 0.03 | 0.125 | 1 |
| 9-1862936 | 0.5 | 0.125 | 0.25 | 0.03 | 0.125 | 1 |
| E5-1033091 | 0.5 | 0.03 | 0.25 | 0.02 | 0.06 | 1 |
| 9-26422166 | 0.5 | 8 | 0.25 | 0.03 | 0.125 | 1 |
| 9-2642158 | 0.25 | 0.25 | 0.25 | 0.06 | 0.125 | 1 |
| E5-1035775 | 0.5 | > 16 | 0.25 | 0.06 | 0.125 | 1 |
| E5-1029558 | 0.25 | 16 | 0.5 | 0.03 | 0.125 | 1 |
| E5-1038279 | 0.5 | 4 | 0.25 | 0.03 | 0.125 | 1 |
| E5-1039697 | 0.25 | 0.5 | 0.25 | 0.06 | 0.125 | 1 |
| E5-1041979 | 0.5 | 0.25 | 0.5 | 0.03 | 0.125 | 1 |
| E5-1035284 | 0.25 | 0.25 | 0.25 | 0.03 | 0.125 | 1 |
| E5-1030469 | 0.25 | 0.125 | 0.25 | 0.03 | 0.125 | 2 |
| E5-1030472 | 0.5 | 0.25 | 0.5 | 0.06 | 0.125 | 1 |
| E5-1041977 | 0.5 | 16 | 0.5 | 0.03 | 0.125 | 2 |
| E5-1041987 | 0.5 | 16 | 0.25 | 0.03 | 0.125 | 1 |
| E5-1039684 | 0.5 | 16 | 0.25 | 0.06 | 0.125 | 1 |
| E5-1041980 | 0.25 | 0.25 | 0.25 | 0.03 | 0.125 | 1 |
| E5-1033088 | 0.25 | 0.25 | 0.25 | 0.03 | 0.125 | 1 |
| E5-1035277 | 0.5 | 16 | 0.5 | 0.03 | 0.125 | 1 |
| E5-1046096 | 0.5 | 0.5 | 0.5 | 0.06 | 0.125 | 1 |
| E5-1046085 | 0.5 | 8 | 0.5 | 0.06 | 0.125 | 2 |
| 9-2625962 | 0.5 | 0.25 | 0.5 | 0.03 | 0.125 | 1 |
| E5-1043668 | 0.25 | 1 | 0.25 | 0.06 | 0.25 | 1 |
| E5-1048428 | 0.25 | 0.5 | 0.25 | 0.06 | 0.25 | 1 |
| E5-1047924 | 0.5 | 0.25 | 0.5 | 0.03 | 0.125 | 1 |
| E5-1047606 | 0.5 | 8 | 0.5 | 0.03 | 0.125 | 1 |
| E5-1046070 | 0.25 | 0.25 | 0.5 | 0.03 | 0.125 | 1 |
| E5-1046298 | 0.25 | 0.125 | 0.25 | 0.03 | 0.125 | 1 |
| E5-1046296 | 0.5 | 0.125 | 1 | 0.03 | 0.125 | 1 |
| E5-1046297 | 0.5 | 0.125 | 0.25 | 0.06 | 0.125 | 1 |
| E5-1043184 | 0.5 | 16 | 0.5 | 0.03 | 0.125 | 1 |
| E5-1038286 | 0.25 | 0.25 | 0.5 | 0.06 | 0.125 | 1 |
| E5-1037958 | 0.5 | 16 | 0.5 | 0.06 | 0.125 | 1 |
| E5-1037971 | 0.25 | 0.25 | 0.25 | 0.03 | 0.125 | 1 |
| E5-1033076 | 0.5 | 0.25 | 0.25 | 0.03 | 0.125 | 1 |
| E5-1033076 | 0.5 | 0.25 | 0.25 | 0.03 | 0.125 | 1 |
| E5-1029252 | 0.25 | 0.25 | 0.5 | 0.03 | 0.125 | 1 |
| E5-1030440 | 0.25 | 0.25 | 0.25 | 0.06 | 0.06 | 1 |
| E5-1030482 | 0.125 | 16 | 0.25 | 0.02 | 0.06 | 1 |
| E5-1046074 | 0.25 | 0.125 | 0.25 | 0.06 | 0.125 | 1 |
| E5-1048204 | 0.25 | 0.25 | 0.25 | 0.03 | 0.06 | 2 |
| E5-1048670 | 0.125 | 0.5 | 0.5 | 0.06 | 0.125 | 2 |
| E5-1046039 | 0.25 | 0.25 | 0.25 | 0.06 | 0.125 | 1 |
| E5-1045179 | 0.25 | 0.25 | 0.25 | 0.06 | 0.125 | 1 |
| E5-1046723 | 0.25 | 0.5 | 0.25 | 0.06 | 0.125 | 2 |
|  | **ATx201** | **Rifampicin** |  |  |  |  |
| RN422 | 0.25 | 0.004 |  |  |  |  |

# Table S6: Mutation rates (µ) and frequencies of resistant mutants (*F*) for rifampicin in *S. aureus* strains

| Strain |  | Rifampicin (16 µg/mL) | | | |
| --- | --- | --- | --- | --- | --- |
|  |  | *F* |  | µ | 95% CI |
| MRSA 43484 |  | 4.28$\times$10^–8^ |  | 5.66$\times$10^–9^ | 4.89–6.54$\times$10^–9^ |
| RN4220 |  | 1.28$\times$10^–8^ |  | 1.39$\times$10^–9^ | 1.60–1.82$\times$10^–9^ |
| RN4220 Δ*mutS* |  | 4.94$\times$10^–7^ |  | 5.11$\times$10^–8^ | 4.72–5.55$\times$10^–8^ |

# Table S7: Baseline demographics per regimen group

|  | **Once-daily (N=18)** | **Twice-daily (N=18)** |
| --- | --- | --- |
| **Gender (N (% of total))** | | |
| Female | 5 (27.8%) | 13 (72.2%) |
| Male | 13 (72.2%) | 5 (27.8%) |
| **Ethnicity (N (% of total))** | | |
| Asian | 1 (5.6%) | 2 (11.1%) |
| Caucasian | 17 (94.4%) | 16 (88.9%) |
| **Age, yr (± SD)** | 31.56 (± 9.85) | 34.17 (± 14.05) |
| **Weight (kg)** | 69.32 (±12.01) | 74.08 (± 18.03) |
| **Height (cm)** | 173.78 (± 8.67) | 170.44 (± 8.45) |
| **BMI (kg/m^2^)** | 22.86 (± 2.86) | 25.34 (± 5.44) |

# Table S8: Summary of AEs by Organ class, relationship to treatment, outcome, and detailed description of administration site conditions. The split-body design of the clinical trial precludes assessing systemic AEs per treatment regimen.

| SOC/ Diagnosis/ Relationship | Outcome | # of AEs emerged during treatment | qd | bid | Total | % of all AEs (51) | % of SAF (43) |
| --- | --- | --- | --- | --- | --- | --- | --- |
| Blood and lymphatic system disorders |  |  | **2** | **0** | **2** | 3.9 | 9.1 |
| *Leukocytosis* |  |  |  |  |  |  |  |
| Not Related | Recovered/Resolved | 1/1 | 1 | 0 | 1 |  |  |
| Unlikely | Recovering/Resolving | 1/1 | 1 | 0 | 1 |  |  |
| Eye disorders |  |  | **1** | **0** | **1** | 2.0 | 4.6 |
| *Eye pruritus* |  |  |  |  |  |  |  |
| Unlikely | Recovered/Resolved | 1/1 | 1 | 0 | 1 |  |  |
| Gastrointestinal disorders |  |  | **0** | **7** | **7** | 13.7 | 31.9 |
| *Abdominal discomfort* |  |  |  |  |  |  |  |
| Not Related | Recovered/Resolved | 1/1 | 0 | 1 | 1 |  |  |
| *Abdominal pain, upper* |  |  |  |  |  |  |  |
| Not Related | Recovered/Resolved | 1/1 | 0 | 1 | 1 |  |  |
| *Diarrhoea* |  |  |  |  |  |  |  |
| Not Related | Recovered/Resolved | 2/3 | 0 | 3 | 3 |  |  |
| *Vomiting* |  |  |  |  |  |  |  |
| Not Related | Recovered/Resolved | 1/2 | 0 | 2 | 2 |  |  |
| General disorders and administration site conditions |  |  | **2** | **7** | **9** | 17.6 | 41 |
| *Application site erythema* |  |  |  |  |  |  |  |
| Probably | Recovered/Resolved | 1/1 | 1 | 0 | 1 |  |  |
| *Application site pruritus* |  |  |  |  |  |  |  |
| Possibly | Recovered/Resolved | 1/1 | 1 | 0 | 1 |  |  |
| *Burning sensation* |  |  |  |  |  |  |  |
| Probably | Recovered/Resolved | 1/1 | 0 | 1 | 1 |  |  |
| Related | Recovered/Resolved | 1/1 | 0 | 1 | 1 |  |  |
| Unlikely | Recovered/Resolved | 1/1 | 0 | 1 | 1 |  |  |
| *Fatigue* |  |  |  |  |  |  |  |
| Unlikely | Recovered/Resolved | 1/1 | 0 | 1 | 1 |  |  |
| *Feeling hot* |  |  |  |  |  |  |  |
| Unlikely | Recovered/Resolved | 1/1 | 0 | 1 | 1 |  |  |
| *Influenza like illness* |  |  |  |  |  |  |  |
| Not Related | Recovered/Resolved | 1/1 | 0 | 1 | 1 |  |  |
| *Pyrexia* |  |  |  |  |  |  |  |
| Not Related | Recovered/Resolved | 1/1 | 0 | 1 | 1 |  |  |
| Infections and infestations |  |  | **2** | **2** | **4** | 7.8 | 18.2 |
| *Conjunctivitis* |  |  |  |  |  |  |  |
| Not Related | Recovered/Resolved | 1/1 | 1 | 0 | 1 |  |  |
| *Nasopharyngitis* |  |  |  |  |  |  |  |
| Not Related | Recovered/Resolved | 1/1 | 1 | 0 | 1 |  |  |
| *Tonsillitis* |  |  |  |  |  |  |  |
| Not Related | Recovered/Resolved | 1/1 | 0 | 1 | 1 |  |  |
| *Urinary tract infection* |  |  |  |  |  |  |  |
| Not Related | Recovered/Resolved | 0/1 | 0 | 1 | 1 |  |  |
| Metabolism and nutrition disorders |  |  | **0** | **1** | **1** | 2.0 | 4.6 |
| *Hyponatremia* |  |  |  |  |  |  |  |
| Unlikely | Recovered/Resolved | 1/1 | 0 | 1 | 1 |  |  |
| Nervous system disorders |  |  | **3** | **3** | **6** | 11.8 | 27.4 |
| *Headache* |  |  |  |  |  |  |  |
| Not Related | Recovered/Resolved | 3/3 | 3 | 0 | 3 |  |  |
| Unlikely | Recovered/Resolved | 2/2 | 0 | 2 | 2 |  |  |
| *Migraine* |  |  |  |  |  |  |  |
| Not Related | Recovered/Resolved | 1/1 | 0 | 1 | 1 |  |  |
| Psychiatric disorders |  |  | **1** | **0** | **1** | 2.0 | 4.6 |
| *Insomnia* |  |  |  |  |  |  |  |
| Not Related | Recovered/Resolved | 1/1 | 1 | 0 | 1 |  |  |
| Reproductive system and breast disorders |  |  | **1** | **0** | **1** | 2.0 | 4.6 |
| *Dysmenorrhoea* |  |  |  |  |  |  |  |
| Not Related | Recovered/Resolved | 0/1 | 1 | 0 | 1 |  |  |
| Respiratory, thoracic and mediastinal disorders |  |  | **0** | **1** | **1** | 2.0 | 4.6 |
| *Oropharyngeal pain* |  |  |  |  |  |  |  |
| Not Related | Recovered/Resolved | 1/1 | 0 | 1 | 1 |  |  |
| Skin and subcutaneous tissue disorders |  |  | **7** | **11** | **18** | 35.3 | 82.1 |
| *Dermatitis atopic* |  |  |  |  |  |  |  |
| Not Related | Ongoing | 3/3 | 1 | 2 | 3 |  |  |
|  | Recovering/Resolving | 1/1 | 1 | 0 | 1 |  |  |
|  | Recovered/Resolved | 5/5 | 3 | 2 | 5 |  |  |
| Unlikely | Ongoing | 2/2 | 1 | 1 | 2 |  |  |
|  | Recovered/Resolved | 1/1 | 1 | 0 | 1 |  |  |
| Possibly | Recovered/Resolved | 1/1 | 0 | 1 | 1 |  |  |
| *Pruritus* |  |  |  |  |  |  |  |
| Not Related | Ongoing | 1/1 | 0 | 1 | 1 |  |  |
|  | Recovered/Resolved | 1/1 | 0 | 1 | 1 |  |  |
| *Rash* |  |  |  |  |  |  |  |
| Unlikely | Recovered/Resolved | 2/2 | 0 | 2 | 2 |  |  |
| *Skin exfoliation* |  |  |  |  |  |  |  |
| Possibly | Recovered/Resolved | 1/1 | 0 | 1 | 1 |  |  |
| Total |  |  | **19** | **32** | **51** | **100.0** | **232.6** |

| *Administration site conditions per treatment regimen and relationship* | | | |
| --- | --- | --- | --- |
|  |  |  |  |
|  | **ATx201** | **Vehicle** | **Total** |
| **Application site erythema** | **1** | **0** | **1** |
| Probably | 1 | 0 | 1 |
| **Application site pruritus** | **0** | **1** | **1** |
| Possibly | 0 | 1 | 1 |
| **Burning sensations** | **2** | **2** | **4** |
| Unlikely | 0 | 1 | 1 |
| Probably | 1 | 0 | 1 |
| Related | 1 | 1 | 2 |
| **Total** | **3** | **3** | **6** |

Note: AE = Adverse event, qd. = once-daily, SAF = Safety analyzable population, SOC = System organ class, bid = twice-daily.

# Table S9: Summary of modified EASI, lesional VAS and lesional IGA score

|  | **Baseline** | | | | **Day 7** | | | |
| --- | --- | --- | --- | --- | --- | --- | --- | --- |
|  | **Active** | | **Vehicle** | | **Active** | | **Vehicle** | |
|  | **Qd** | **Bid** | **Qd** | **Bid** | **Qd** | **Bid** | **Qd** | **Bid** |
| **modified EASI** | | | | | | | | |
| Mean  (SD) | 8.56  (2.48) | 7.06 (2.15) | 8.72 (2.3) | 7 (2.2) | 7.17 (3.03) | 6.78 (2.21) | 7.06 (2.96) | 6.06 (2.61) |
| Number | 18 | 18 | 18 | 18 | 18 | 18 | 18 | 18 |
| **lesional VAS** | | | | | | | | |
| Mean  (SD) | 4.37 (3.07) | 4.68 (3.06) | 4.5 (3.11) | 4.51 (3.25) | 2.53  (2.53) | 3.71 (2.79) | 3.25 (2.81) | 3.28 (3.02) |
| Number | 18 | 18 | 18 | 18 | 18 | 18 | 18 | 18 |
| **lesional IGA** | | | | | | | | |
| Score 1, % (N) | 0 (0) | 5.6 (1) | 0 (0) | 5.6 (1) | 11.1 (2) | 16.7 (3) | 5.6 (1) | 11.1 (2) |
| Score 2, % (N) | 5.6 (1) | 16.7 (3) | 5.6 (1) | 27.8 (5) | 22.2 (4) | 5.6 (1) | 27.8 (5) | 11.1 (2) |
| Score 3, %  (N) | 55.6 (10) | 61.1 (11) | 61.1 (11) | 50.0 (9) | 38.9 (7) | 55.6 (10) | 44.4 (8) | 61.1 (11) |
| Score 4, % (N) | 38.9 (7) | 16.7 (3) | 33.3 (6) | 16.7 (3) | 27.8 (5) | 22.2 (4) | 22.2  (4) | 16.7 (3) |
| **lesional IGA** |  |  |  |  |  |  |  |  |
| 0-1, %  (N) | 0 (0) | 5.6 (1) | 0 (0) | 5.6 (1) | 11.1 (2) | 16.7 (3) | 5.6 (1) | 11.1 (2) |
| Score 2, % (N) | 5.6 (1) | 16.7 (3) | 5.6 (1) | 27.8 (5) | 22.2 (4) | 5.6 (1) | 27.8 (5) | 11.1 (2) |
| Score > 2, % (N) | 94.4  (17) | 77.8  (14) | 94.4  (17) | 66.7  (12) | 66.7  (12) | 77.8  (14) | 66.7  (12) | 77.8  (14) |

Bid = twice daily, EASI = Eczema Area and Severity Index, IGA = Investigator Global assessment, N = Number , Qd = once daily, VAS = visual analog scale

# Table S10: MICs of ATx201 in culturable strains isolated from patients at Day 7

| **Isolate Day** | **Subject** | **Dose** | **MIC [µg/mL]** |
| --- | --- | --- | --- |
| D7 | C01 | bid | 0.06 |
| D7 | C02 | qd | 0.06 |
| D7 | C03 | qd | 0.25 |
| D7 | C04 | bid | 0.06 |
| D7 | C05 | bid | 0.125 |
| D7 | C06 | qd | 0.25 |
| D7 | C08 | bid | 0.5 |
| D7 | C11 | qd | 0.25 |
| D7 | C12 | qd | 0.06 |
| D7 | C13 | bid | 0.5 |
| D7 | C14 | qd | 0.125 |
| D7 | C16 | qd | 0.25 |
| D7 | C17 | bid | 0.5 |
| D7 | C18 | qd | 0.25 |
| D7 | C20 | qd | 0.5 |
| D7 | C22 | qd | 0.125 |
| D7 | C23 | bid | 0.25 |
| D7 | C26 | qd | 0.25 |
| D7 | C27 | bid | 0.25 |
| D7 | C28 | bid | 0.25 |
| D7 | C30 | qd | 0.25 |
| D7 | C32 | qd | 0.06 |
| D7 | C34 | qd | 0.25 |
| D7 | C38 | qd | 0.25 |
| D7 | C39 | qd | 0.125 |
| D7 | C43 | bid | 0.125 |
| QC | ATCC 29213 |  | 0.125 – 0.25 |

Note: Bid = twice-daily, QC = quality control, qd = once-daily
